# Life-death trade-offs: HSV-1 ICP27 differentially modulates intrinsic apoptotic signaling in epithelial and neuron-like cells

**DOI:** 10.3389/fmicb.2026.1849379

**Published:** 2026-06-15

**Authors:** Chiara Nordi, Anna Caproni, Davide Barboni, Davide Proietto, Martina Facchini, Riccardo Fontana, Mattia Buratto, Martina Catani, Peggy Marconi

**Affiliations:** 1 Laboratory of Microbiology, Department of Environmental and Prevention Sciences, University of Ferrara, Ferrara, Italy; 2 Laboratory of Analytical Chemistry, Department of Chemical, Pharmaceutical and Agricultural Sciences, University of Ferrara, Ferrara, Italy; 3 Laboratory of Microbiology, Department of Chemical, Pharmaceutical and Agricultural Sciences, University of Ferrara, Ferrara, Italy; 4 LTTA Laboratory for Advanced Therapies, Technopole of Ferrara, Ferrara, Italy

**Keywords:** apoptosis, HSV-1, hTert RPE-1, ICP27, SH-SY5Y

## Abstract

Herpes simplex virus type 1 (HSV-1) is a well-established model to investigate virus-host interactions due to its ability to modulate cell fate and establish lifelong persistence. Among the immediate-early genes, the multifunctional regulatory protein ICP27, encoded by the *UL54* gene, plays a central role in viral mRNA processing, nuclear export, and reprogramming host gene expression. This study investigates the role of ICP27 in modulating the intrinsic apoptotic pathway in two cell models representative of different stages of infection: hTert RPE-1 epithelial cells, modeling the lytic replication, and SH-SY5Y neuroblastoma cells, used as a neuron-like model to approximate a neuronal microenvironment. To dissect the specific contribution of HSV-1 ICP27 to apoptosis regulation, cells infected with wild-type HSV-1 (HSV-1 w.t.) were compared with a viral vector lacking ICP27 (HSV-1ΔICP27). A multi-layered experimental approach was employed to characterize infection-induced cellular responses. Analyses were conducted using RT-qPCR for gene quantification, western blotting for protein evaluation, enzymatic assays to measure caspase activity, flow cytometry to determine reactive oxygen species (ROS) production and mitochondrial functionality, and quantitative proteomic approaches for the global characterization of infection-induced protein changes. ICP27 is associated with the modulation of apoptotic signal in a cell type-dependent manner. In epithelial cells, the presence of ICP27 is linked to intensified pro-apoptotic markers, including activation of caspase-9 and effector caspases (3/7), increased ROS generation, altered mitochondrial function, and elevated Bax/Bcl-2 ratio, indicative of mitochondrial pathway activation. Conversely, in neuron-like cells, ICP27 expression appears to correlate with reduced apoptotic signaling, characterized by lower caspase activity, limited oxidative stress, and preserved mitochondrial integrity. Overall, our findings indicate that ICP27 differentially modulates apoptotic signaling depending on the cellular context, enhancing pro-apoptotic features in epithelial cells while limiting intrinsic pathway activation in neuron-like cells. This dual behavior underscores HSV-1 adaptability across microenvironments, with implications for viral pathogenesis and therapeutic targeting.

## 1 Introduction

Apoptosis is a highly regulated form of programmed cell death that allows the organism to eliminate damaged, infected, or unnecessary cells without inducing inflammation (Zayani et al., 2025). Morphologically, apoptosis is characterized by an ordered sequence of events, including chromatin condensation, nuclear fragmentation, membrane blebbing, and the formation of rapidly phagocytosed apoptotic bodies, in stark contrast to the uncontrolled lysis typical of necrosis (Fulda et al., 2010; Krysko et al., 2008; Fink and Cookson, 2005; Elmore, 2007; Doonan and Cotter, 2008).

**Figure 1:**
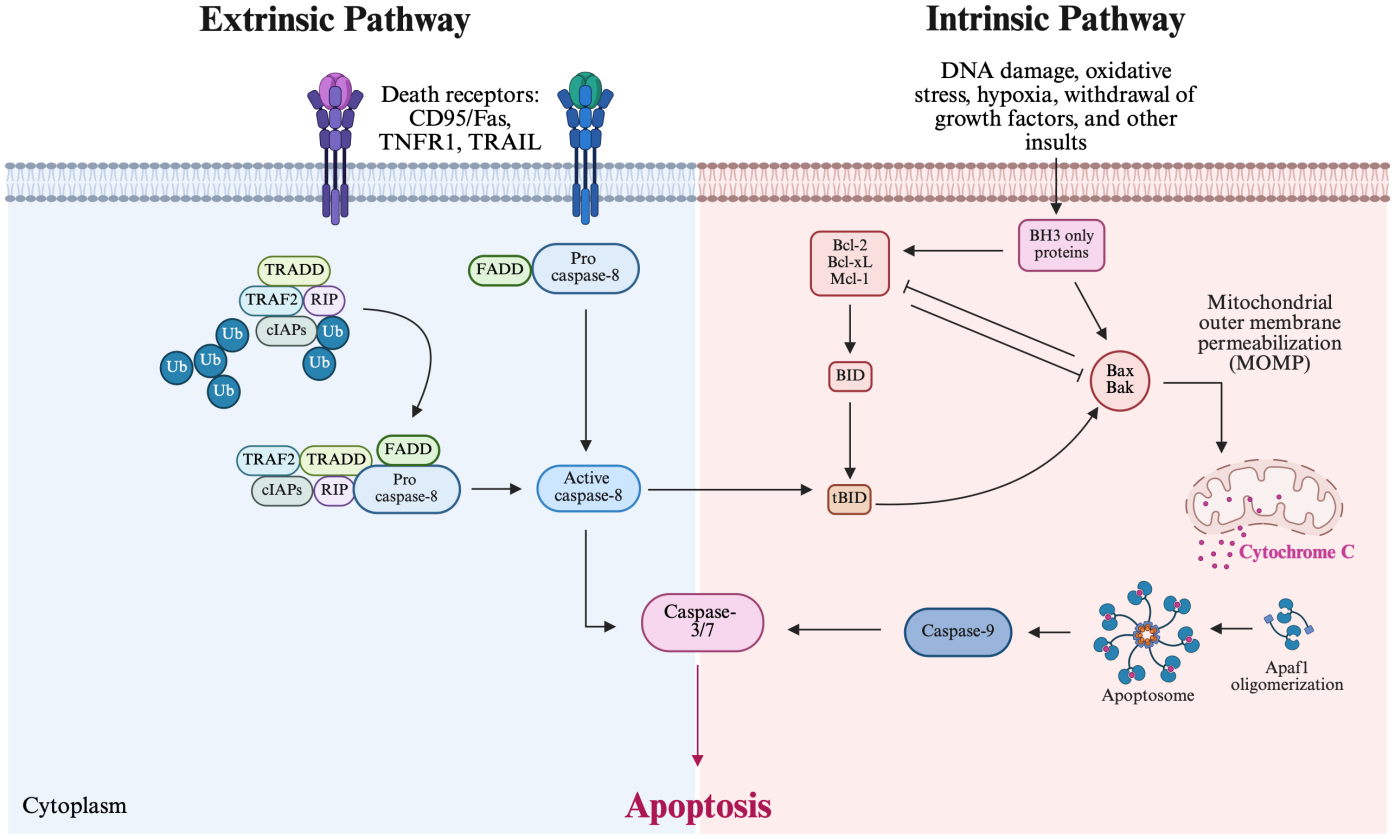
Representation of key proteins involved in the extrinsic and intrinsic pathways of apoptosis. Image created with BioRender.com.

At the molecular level, pro-apoptotic signals converge mainly on two cascades: the extrinsic pathway, mediated by death receptors (e.g., Fas, TNFR1, TRAIL receptors) (Kumar et al., 2005; Nair et al., 2014; Lavrik, 2014; Guerrache and Micheau, 2024), and the intrinsic or mitochondrial pathway, activated by intracellular stresses such as DNA damage, oxidative stress or trophic factor deprivation (Wu and Bratton, 2013; Rodríguez-González et al., 2024; Johnson and Sarosiek, 2024). The intrinsic pathway is finely regulated by pro- and anti-apoptotic Bcl-2 family proteins, adaptors such as Apaf-1 and by mitochondrial factors including cytochrome c, Smac/DIABLO, and AIF (Tsujimoto, 1998; Qian et al., 2022; Singh et al., 2019; Shakeri et al., 2017), whereas both pathways ultimately lead to the sequential activation of initiator and executor caspases (Parrish et al., 2013; Nadendla et al., 2025; Zhou et al., 2015; Kim et al., 2005) (Figure 1). Dysregulation of these pathways is implicated in many pathological conditions, including tumors, autoimmune diseases, and neurodegenerative diseases, underscoring the clinical relevance of their modulation (Mustafa et al., 2024; Orning and Lien, 2021).

In the context of viral infections, apoptosis represents an important host defense mechanism, that limits the production of new viral particles by eliminating infected cells at early stages (Barber, 2001; Orzalli and Kagan, 2017; Kvansakul, 2017). Numerous viruses, however, have evolved strategies to selectively manipulate these pathways, suppressing cell death when efficient virion production is required and, conversely, inducing it when it promotes immune dissemination or evasion. Herpes simplex virus type 1 (HSV-1) represents a paradigmatic example of viral plasticity, as it encodes multiple proteins capable of modulating both intrinsic and extrinsic apoptotic pathways (Aubert and Blaho, 2001; Guo et al., 2022; Kather et al., 2010; You et al., 2017). Several studies have shown that HSV-1 can activate both the extrinsic pathway, for instance by modulating Fas/FasL and TRAIL receptors (Marino-Merlo et al., 2023), and the intrinsic pathway, through mitochondrial stress, increased production of reactive oxygen species (ROS), cytochrome c release (Papaianni et al., 2015; Sorouri et al., 2022; Whitaker and Cook, 2021). At the same time, HSV-1 expresses multiple anti-apoptotic factors, such as Us3 serine/threonine kinase, the large subunit of ribonucleotide reductase UL39 (Benetti et al., 2003; Cartier et al., 2003), and other immediate-early proteins, which act at various nodes of the caspase cascade to prevent premature cell death and thereby promote productive infection or stabilize latency in neurons (Sanfilippo and Blaho, 2006; Boutell and Everett, 2003; Jerome et al., 2001; Dufour et al., 2011; Gillis et al., 2009; He et al., 2021; Goldsmith et al., 1998; Koyanagi and Kawaguchi, 2020; Benedict et al., 2003; Šedý et al., 2008).

Among HSV-1 immediate-early proteins, ICP27 is a multifunctional regulator essential for late viral gene expression and for the remodelling of host signaling pathways (Sandri-Goldin, 2008; Koffa, 2001). The available literature indicates that the role of ICP27 on apoptosis is complex and apparently context-dependent. Studies conducted with ICP27-deficient viruses have shown that its absence increases cellular susceptibility to apoptosis, suggesting a cytoprotective function in the early stages of infection (Sanfilippo et al., 2004; Aubert et al., 2001). Other works, on the contrary, have shown that ICP27 can promote mitochondrial apoptosis, for example by promoting Bax translocation to mitochondria through interference with 14-3-3θ binding (Kim et al., 2017), and by modulating kinases such as p38 MAPK and JNK (Gillis et al., 2009; Hargett et al., 2005). Overall, these observations suggest that ICP27 is associated with both pro- and anti-apoptotic effects depending on cellular context and infection stage.

Despite these findings, it remains unclear whether ICP27 exerts differential effects on intrinsic apoptotic signaling across distinct cellular environments. In particular, the extent to which ICP27 modulates apoptosis in epithelial versus neuron-like cells remains insufficiently characterized. In this study, SH-SY5Y cells were used as a neuron-like model to approximate a neuronal microenvironment, although they do not fully recapitulate the physiology of primary neurons. Addressing this question is essential to understanding how HSV-1 balances cell death and survival to optimize viral replication, dissemination, and long-term persistence.

## 2 Materials and methods

### 2.1 Cell lines

Vero cells (ATCC CCL-81) and 7b cells (Vero-derived cells stably expressing HSV-1 immediate-early genes *RS1*/ICP4 and *UL54*/ICP27) were used for viral propagation. Both cell lines were maintained in Dulbecco’s Modified Eagle’s Medium (DMEM) supplemented with 10% heat-inactivated fetal bovine serum (FBS), 100 U/mL penicillin, 100 μg/mL streptomycin, and 2 mM L-glutamine (all from Euroclone, Milano, Italy). 7b cells were cultured in the presence of 100 μg/mL G418 (Geneticin solution; Euroclone, Milano, Italy) to maintain transgene selection.

hTert RPE-1 cells (ATCC CRL-400), an immortalized human retinal pigment epithelial cell line expressing human telomerase reverse transcriptase (hTert), were cultured in DMEM/F12 (Euroclone, Milan, Italy) supplemented with 10% heat-inactivated FBS, 100 U/mL penicillin, 100 μg/mL streptomycin, 2 mM L-glutamine, and 0.01 mg/mL hygromycin B (Merck Life Science, Milano, Italy).

SH-SY5Y cells (ATCC CRL-2266), a human neuroblastoma-derived neuron-like cell line, were maintained in DMEM/F12 supplemented with 15% heat-inactivated FBS, 100 U/mL penicillin, 100 μg/mL streptomycin, and 2 mM L-glutamine.

All cell lines were cultured at 37 °C in a humidified 5% CO₂ atmosphere. Cells were regularly tested for mycoplasma contamination and used within 15 passages.

### 2.2 Plasmid and virus construction

The HSV-1ΔICP27 mutant virus was generated by recombineering-mediated deletion of the *UL54* gene (nucleotides 113,323–115,743; BamHI-SacI region) from a bacterial artificial chromosome (BAC) containing the full-length HSV-1 strain F genome (NCBI: txid10304), inserted in the UL3-UL4 intergenic region. Recombineering was performed in SW102 *Escherichia coli* using a two-step galK selection strategy.

The *UL54* genomic fragment was amplified and cloned into pBSSK (Stratagene, San Diego, CA, United States) to generate the pBICP27 expression plasmid.

Correct genomic modification was verified by PCR amplification of the *UL54* locus and functional deletion was confirmed by absence of ICP27 protein expression by western blot.

HSV-1ΔICP4 was used as a replication-defective control virus to exclude ICP4-dependent effects. This herpetic vector was kindly provided by J.C. Glorioso.

### 2.3 Viral propagation and titration

HSV-1 strain F stocks were prepared in Vero cells infected at a multiplicity of infection (MOI) of 0.01. HSV-1ΔICP27 and HSV-1ΔICP4 stocks were generated in 7b cells at 0.03 MOI as previous described (Marconi et al., 1996; Warden et al., 2011; Fraefel et al., 2011; Marconi et al., 2015).

Following complete cytopathic effect (CPE), cells and supernatants were collected and clarified by centrifugation (2,000 rpm, 10 min). Cell pellets underwent three freeze–thaw cycles (−80 °C/37 °C) and sonication to release intracellular virions. Supernatants were pooled and concentrated by ultracentrifugation (20,000 rpm, 30 min) using JA20 rotor (Beckman Coulter, Brea, CA, United States).

Viral aliquots were stored at −80 °C and thawed only once prior to use.

Viral titers were determined by plaque assay on Vero or 7b cells. After 1-h adsorption, inoculum was replaced with DMEM containing 1% methylcellulose. Plaques were visualized 72 h post-infection by crystal violet staining and titers expressed as PFU/mL. All reagents and consumables were procured from Merck Life Science S.r.l., Milano, Italy.

### 2.4 Infection conditions

hTert RPE-1 and SH-SY5Y cells were infected with HSV-1 wild-type strain F or HSV-1ΔICP27 at 3 MOI.

An MOI of 3 was selected to ensure synchronous infection while minimizing excessive cytopathic effects during early time points.

Where indicated, HSV-1ΔICP4 was included as control.

Infections were performed for 2, 4, 8, and 24 h for downstream analyses, including MTT assay and LDH assay. Samples were collected at 4 and 8 h.p.i. for qPCR, caspase activity, ROS, and mitochondrial analyses. For western blotting and proteomic analyses, cells were exclusively collected at 8 h.p.i. Untreated cells served as controls.

### 2.5 Cell proliferation and viability assay

Cell viability following infection with HSV-1 w.t., HSV-1ΔICP27 or HSV-1ΔICP4 was assessed using the colorimetric MTT assay (3-(4,5-dimethylthiazol-2-yl)-2,5-diphenyltetrazolium bromide) Cell Proliferation Kit I (Roche, Monza, Italy). This assay relies on the ability of metabolically active cells to convert the yellow tetrazolium salt (MTT) into insoluble purple formazan crystals through mitochondrial enzymatic activity, providing an indirect measure of cell viability.

hTert RPE-1 and SH-SY5Y cells were seeded in 96-well plates (1 × 10^4^ cells per well) and allowed to adhere overnight prior to infection. Each condition was analyzed in technical triplicate. A cell-free control (blank) containing complete culture medium was included on each assay plate to determine background absorbance.

After the designated infection time points, 10 μL of MTT reagent (5 mg/mL) was added to each well and plates were incubated for 4 h at 37 °C. Formazan crystals were then solubilized by addition of solubilization buffer (10% SDS in 0.01 M HCl) followed by overnight incubation at 37 °C.

Absorbance was measured at 570 nm with a reference wavelength of 600 nm using the GloMax Discover microplate reader (Promega, Milano, Italy). Values were normalized to untreated controls after subtraction of background signal.

Percentage viability was calculated as follows:


$$\text{Percentage viability}=\frac{\begin{array}{l} \text{Absorbance of Infected Cells}-\\ \text{Absorbance of Blank} \end{array}}{\begin{array}{l} \text{Absorbance of Untreated Cells}-\\ \text{Absorbance of Blank} \end{array}}\times 100$$

### 2.6 Cell cytotoxicity assay

Plasma membrane damage induced by infection with wild-type HSV-1 and HSV-1ΔICP27 was evaluated using the LDH-Glo Cytotoxicity Assay (Promega, Milan, Italy). This bioluminescent method measures the activity of the enzyme lactate dehydrogenase (LDH) released into the culture medium following cell membrane damage.

hTert RPE-1 and SH-SY5Y cell lines were cultured in 96-well plates (1 × 10^4^ cells per well) and allowed to adhere overnight prior to infection. Each experimental condition was analyzed in technical triplicate. At each established post-infection time point, 2 μL of supernatant was taken from each well, diluted 1:100 in LDH Storage Buffer (200 mM Tris–HCl, pH 7.3, 10% glycerol, 1% bovine serum albumin, all reagents were procured from Merck Life Science S.r.l., Milano, Italy) and stored at −20 °C to preserve enzyme activity.

Control conditions included a medium-only blank, untreated cells (baseline LDH release), and a maximum LDH release control obtained by treating cells with 10% Triton^®^ X-100 for 10–15 min prior to supernatant collection.

For detection, samples were incubated for 1 h at room temperature with LDH Detection Enzyme Mix and Reductase Substrate according to the manufacturer’s instructions. Luminescence was measured using the GloMax Discover microplate reader (Promega, Milano, Italy), and values were normalized to the maximum LDH release control.


$$\text{Percentage cytotoxicity}=\frac{\begin{array}{l} \text{Experimental}\;\mathrm{LDH}\;\text{Release}-\\ \text{Medium Background} \end{array}}{\begin{array}{l} \text{Maximum}\;\mathrm{LDH}\;\text{Release Control}-\\ \text{Medium Background} \end{array}}\times 100$$

### 2.7 Total RNA extraction and real-time PCR

Following the infection period in 6-well plates (1 × 10^6^ cells per well), cells were harvested, washed with 1X phosphate-buffered saline (PBS: 137 mM NaCl, 2.7 mM KCl, Na_2_HPO_4_ 10 mM, KH_2_PO_4_ 1.8 mM, pH 7.35), lysed in TRIzol reagent (Merck Life Science S.r.l., Milano, Italy), and total RNA extracted according to the manufacturer’s instructions: chloroform phase separation, isopropanol precipitation of RNA, and ethanol wash (all reagents were procured from Merck Life Science S.r.l., Milano, Italy).

RNA concentration and purity were determined spectrophotometrically (260/280 nm), and integrity was verified prior to reverse transcription.

Complementary DNA (cDNA) was synthesized from equal RNA amounts using the High-Capacity cDNA Reverse Transcription Kit (Applied Biosystems, Monza, Italy) with optimized thermal cycling conditions, according to the manufacturer’s instructions.

Real-time quantitative PCR was performed in technical triplicate using the CFX96 Touch Real-Time PCR Detection System (Bio-Rad, Segrate, Italy) and PowerUp SYBR Green Master Mix (Applied Biosystems, Monza, Italy). Each run included experimental samples and no-template controls (NTCs). Relative gene expression was calculated using the comparative 2^−ΔΔCt^ method, normalized to the housekeeping genes GAPDH and 18S rRNA, and expressed relative to untreated controls. Amplification specificity was confirmed by melt curve analysis.

Primer sequences are reported in Table 1.

**Table 1:** Forward and reverse primer sequences used for real-time PCR.

Gene	Primer forward	Primer reverse
Caspase-3	GGAAGCGAATCAATGGACTCT	GCATCGACATCTGTACCAGAC
Caspase-7	CGGAACAGACAAAGATGCCGAG	AGGCGGCATTTGTATGGTCCTC
Caspase-8	AGAAGAGGGTCATCCTGGGAGA	TCAGGACTTCCTTCAAGGCTGC
Caspase-9	GTTTGAGGACCTTCGACCAGCT	CAACGTACCAGGAGCCACTCTT
Bax	TCAGGATGCGTCCACCAAGAAG	TGTGTCCACGGCGGCAATCATC
Bcl-2	ATCGCCCTGTGGATGACTGAGT	GCCAGGAGAAATCAAACAGAGGC
GAPDH	GGTGTGAACCATGAGAAGTA	GAGTCCTTCCACGATACCAA
18 s	GTAACCCGTTGAACCCCATT	CCATCCAATCGGTAGTAGCG

### 2.8 Total protein extraction and western blot

After the incubation period in 6-well plates (1 × 10^6^ cells per well), infected and untreated cells were harvested, washed with 1X PBS, and collected by centrifugation. Cell pellets were lysed in ice-cold RIPA buffer (50 mM Tris–HCl pH 8, 1% Triton X-100, 0.5% NP-40, 10 mM *β*-mercaptoethanol, 4% glycerol; all reagents from Merck Life Science S.r.l., Milano, Italy) supplemented with protease inhibitors (Roche, Monza, Italy). Lysates were clarified by centrifugation (14,000 rpm, 10 min, 4 °C), and supernatants containing total proteins were collected and stored at −20 °C for analysis.

Protein concentration was determined using the BCA (bicinchoninic acid) Protein Assay Kit (Thermo Fisher Scientific Italia, Segrate, Italy) and measured at 562 nm with the Glomax Discover microplate reader (Promega, Milano, Italy).

For western blot analysis, 30 μg of total protein per sample was separated on 10% TGX Stain-Free FastCast Acrylamide gels (Bio-Rad, Segrate, Italy) and transferred onto nitrocellulose membranes using the Trans-Blot Turbo system with RTA Transfer Kit (Bio-Rad, Segrate, Italy). Membranes were blocked in TBST containing 5% non-fat dry milk for 1 h at room temperature and incubated with primary antibodies (Table 2) overnight at 4 °C or for 1 h at room temperature, according to antibody specifications. After washing, membranes were incubated with horseradish peroxidase-conjugated secondary antibodies (Table 2) for 1 h at room temperature.

**Table 2:** List of primary and secondary antibodies employed for western blot analysis, including relevant working dilutions, sources, and catalog numbers.

Antibody	Dilution	Source	Identifier
Mouse monoclonal anti-ICP27 antibody	1:1000	Virusys	Cat. No. P1113
Caspase-8 (1C12) mouse monoclonal antibody	1:1000	Cell Signaling Technology	Cat. No. 9746 T
Caspase 9 (C9) mouse monoclonal antibody	1:1000	Cell Signaling Technology	Cat. No. 9508 T
Polyclonal goat anti-mouse immunoglobulins/HRP	1:2000	Dako	Cat. No. P0447
Polyclonal goat anti-rabbit immunoglobulins/HRP	1:2000	Dako	Cat. No. P0448

Protein bands were detected using the ECL NeoPRO Femto chemiluminescence detection kit (NeoBiotech, Nanterre, France) and visualized with the ChemiDoc MP Imaging System (Bio-Rad). Band intensities were quantified using Image Lab software (v6.0.0, Bio-Rad, Segrate, Italy) and normalized to total protein loading using stain-free technology.

### 2.9 Caspase-3/7 activity assay

Caspase-3/7 activities were measured using the Caspase-Glo 3/7 Assay (Promega, Milano, Italy), a luminescent assay where DEVD-proluciferin substrate is cleaved by caspases, producing glow-type luminescence proportional to enzyme activity.

hTert RPE-1 and SH-SY5Y cells were seeded in 96-well plates (1 × 10^4^ cells per well) and infected as described above. At the indicated time points, an equal volume of Caspase-Glo 3/7 Reagent was added directly to each well, mixed for 30 s on an orbital shaker, and incubated at room temperature to allow luminescent signal stabilization. Luminescence was mesured using the GloMax Discover microplate reader (Promega, Milano, Italy). Background signal from cell-free wells was subtracted, and caspase activity was expressed relative to untreated controls. Each condition was analyzed in technical triplicate within three independent biological experiments.


$$\begin{array}{l} \text{Percentage caspase activity}=\\ \frac{\begin{array}{l} \text{Luminescence of Infected Cells}-\\ \text{Luminescence of Blank} \end{array}}{\begin{array}{l} \text{Luminescence of Untreated Cells}-\\ \text{Luminescence of Blank} \end{array}}\times 100 \end{array}$$

### 2.10 Reactive oxygen species production and mitochondrial status

hTert RPE-1 and SH-SY5Y cells were seeded in 6-well plates (1 × 10^6^ cells per well) and infected as described above. At the indicated time points, cells were collected and stained with Live/Dead Fixable Aqua Dead Cell Stain Kit (Thermo Fisher Scientific, Segrate, Italy) to exclude non-viable cells from the analysis.

Intracellular reactive oxygen species (ROS) levels were measured by incubation with CellROX^™^ Green Reagent (5 μM) for 30 min at 37 °C. Basal mitochondrial parameters were assessed in parallel by staining with MitoTracker^™^ Green FM dye (50 μM) to evaluate mitochondrial mass and with tetramethylrhodamine methyl ester (TMRM, 25 nM) in non-quenching mode to assess mitochondrial membrane potential (ΔΨm), each for 30 min at 37 °C. All reagents were procured from Thermo Fisher Scientific Italia, Segrate, Italy.

After staining, cells were washed, resuspended in 1X PBS, and analyzed using a FACS Canto II flow cytometer (BD Biosciences Italia, Milano, Italy). Data were acquired from gated single, live cells after exclusion of debris and doublets. Compensation controls were prepared using BD CompBeads (BD Biosciences Italia, Milano, Italy).

Flow cytometry data were analyzed using FlowJo software version 10.8 (FlowJo LLC, Ashland, OR, United States).

### 2.11 Apoptosis index: Bax/Bcl-2 ratio

The apoptotic index was calculated as the Bax/Bcl-2 ratio using relative mRNA expression levels of the pro-apoptotic gene Bax and the anti-apoptotic gene Bcl-2 determined by real-time qPCR (as described in Section 2.7). For each sample, the normalized expression value of Bax was divided by that of Bcl-2, providing an indicator of the balance between pro- and anti-apoptotic signaling within the intrinsic pathway.

Ratios were calculated from ΔCt-normalized values and expressed relative to untreated controls.

### 2.12 Proteomic analysis

Proteomic analyses were performed on infected and untreated cells collected at 8 h post-infection (h.p.i.). The workflow included protein extraction, purification, enzymatic digestion, and peptide identification by liquid chromatography–tandem mass spectrometry (LC–MS/MS).

All reagents used for proteomics sample preparation and LC–MS/MS analyses—including acetonitrile (ACN), ethanol, trifluoroacetic acid (TFA), formic acid (FA), sodium dodecyl sulfate (SDS), trizma base (TRIS), Tris(2-carboxyethyl)phosphine hydrochloride (TCEP) and chloroacetamide (CAA)—were obtained from Merck Sigma-Aldrich, Darmstadt, Germany. LC–MS-grade trypsin protease was supplied by Thermo Fisher Scientific, Waltham, MA, United States. LC–MS-grade chloric acid and water were purchased from Carlo Erba, Milan, Italy.

#### 2.12.1 Sample preparation

Cell pellets were lysed in a buffer containing 5% SDS, 5 mM TCEP, and 20 mM CAA in 100 mM Tris–HCl (pH 8.0), followed by heating at 95 °C for 10 min with agitation. Lysates were sonicated and clarified by centrifugation (5,000 rpm, 10 min) using a Fresco-17 Centrifuge (Thermo Fisher Scientific, Waltham, MA, United States).

Total protein concentration was determined using the BCA Protein Assay Kit (Thermo Fisher Scientific Italia, Segrate, Italy).

Protein purification was performed using MagReSyn^®^ Hydroxyl magnetic beads (ReSyn Biosciences, Edenvale, South Africa) following the protocol described by Tyanova et al. (2016) with minor modifications. Samples were adjusted to 70% ACN with a protein-to-bead ratio of 1:2 and incubated under agitation. Beads were sequentially washed with 95% ACN and 70% ethanol.

On-bead digestion was carried out in 100 mM Tris–HCl (pH 8.0) using trypsin at an enzyme-to-protein ratio of 1:50 at 37 °C, 1,200 rpm for 16 h. Digestion was quenched by acidification with 1% TFA.

Peptides were purified using Oasis HLB 96-well Plate (3 mg sorbent, Waters, Milford, MA, United States), eluted with 50% ACN/0.1% TFA, dried under vacuum, and reconstituted in 0.1% FA prior to LC–MS/MS analysis.

#### 2.12.2 LC–MS/MS analysis

Tryptic peptides were analyzed using a Vanquish Neo nano-HPLC system coupled online to an Orbitrap Exploris 240 mass spectrometer equipped with a nano-ESI Easy-Spray ion source (Thermo Fisher Scientific, Waltham, MA, United States). Mobile phase A was 0.1% FA in water, and mobile phase B was 80:20 ACN:water with 0.1% FA. Direct injection was performed onto an EASY-Spray PepMap Neo RP-C18 column (150 mm × 75 μm, 2 μm, Thermo Fisher Scientific, Waltham, MA, USA) at a flow rate of 500 nL/min, with the column temperature maintained at 35 °C.

The total runtime was 60 min. The gradient elution was programmed as follows: 1% B for 1.5 min, linear increase to 31% B over 45 min, to 50% in 3.75 min, then to 95% in another 3.75 min. The 95% B phase was maintained for 6 min, followed by a rapid re-equilibration to initial conditions for the next injection.

Peptides were ionized with a spray voltage of +1.9 kV and a capillary temperature of 280 °C. MS full-scan parameters: scan range 350–1,100 Th, RF lens at 70%, Orbitrap resolution of 60,000 (FWHM at m/z 200), automatic maximum injection time, and AGC target of 100%.

MS/MS acquisition was performed in data independent acquisition (DIA) mode. The precursor mass range was 361–1,033 Th, segmented into 56 isolation windows (12 Th each, with 1 Th overlap). The normalized HCD collision energy was set to 28%. Orbitrap resolution for MS2 was 15,000 (FWHM at m/z 200), with auto injection time and AGC target of 1,000%.

Automatic gain control (AGC) targets and maximum injection times were set according to instrument default parameters.

Instrument performance was monitored by blank injections after each batch of samples; a HeLa protein digest (Thermo Fisher Scientific, Waltham, MA, United States) was also injected as a quality control sample at regular intervals.

All raw files were analyzed using Spectronaut v20 (Biognosys AG, Zurich, Switzerland), searching against a library automatically generated by combining *Homo sapiens* and Human herpesvirus 1 (strain 17) Swiss-Prot proteome database (https://www.uniprot.org/, last access 21/08/2025), supplemented with common contaminants and using default settings.

### 2.13 Statistical analysis

All experiments were performed with three independent biological replicates, and technical triplicates were used where applicable. Data are presented as mean ± standard deviation (SD).

Statistical analyses for MTT, LDH, qPCR, western blot densitometry, caspase-3/7 activity, and flow cytometry experiments were performed using one-way or two-way analysis of variance (ANOVA), as appropriate, followed by Dunnett’s *post hoc* test or Šídák’s multiple comparisons test. Analyses were conducted using GraphPad Prism software, version 9.0.0 for macOS (GraphPad Software, San Diego, CA, United States). A *p*-value ≤ 0.05 was considered statistically significant.

For proteomic data, downstream statistical analysis was performed using Perseus software (v2.1.5) (Tyanova et al., 2016). Label-free quantification (LFQ) intensities were log₂-transformed after removal of contaminants and proteins identified with Q-value > 0.05. Proteins detected in at least one experimental group with three valid values out of three replicates were retained for analysis. Missing values were imputed from a normal distribution (downshift 1.8; width 0.3).

Datasets were analyzed separately for each cell line. Differential protein expression was assessed using ANOVA with Benjamini-Hochberg false discovery rate (FDR) correction. Proteins with adjusted Q-value ≤ 0.05 were considered significantly differentially expressed.

Significant proteins were normalized using Z-scores and subjected to unsupervised hierarchical clustering. Pairwise comparisons were additionally performed using two-tailed t-tests with Benjamini-Hochberg FDR correction, applying a log_2_ fold-change threshold ≥ 1 to identify significantly upregulated and downregulated targets.

Protein–protein interaction networks were generated using the STRING database and visualized in Cytoscape. Functional modules were identified using the Markov Cluster Algorithm (MCL) with an inflation parameter of 3.

## 3 Results

### 3.1 Assessment of ICP27 protein expression in the HSV-1ΔICP27 virus

To investigate ICP27 (*UL54*) role in apoptosis modulation, we confirmed absence of ICP27 protein in the HSV-1ΔICP27 mutant virus through western blot. In hTert RPE-1 cells, ICP27 deletion was previously validated (Caproni et al., 2024).

**Figure 2:**
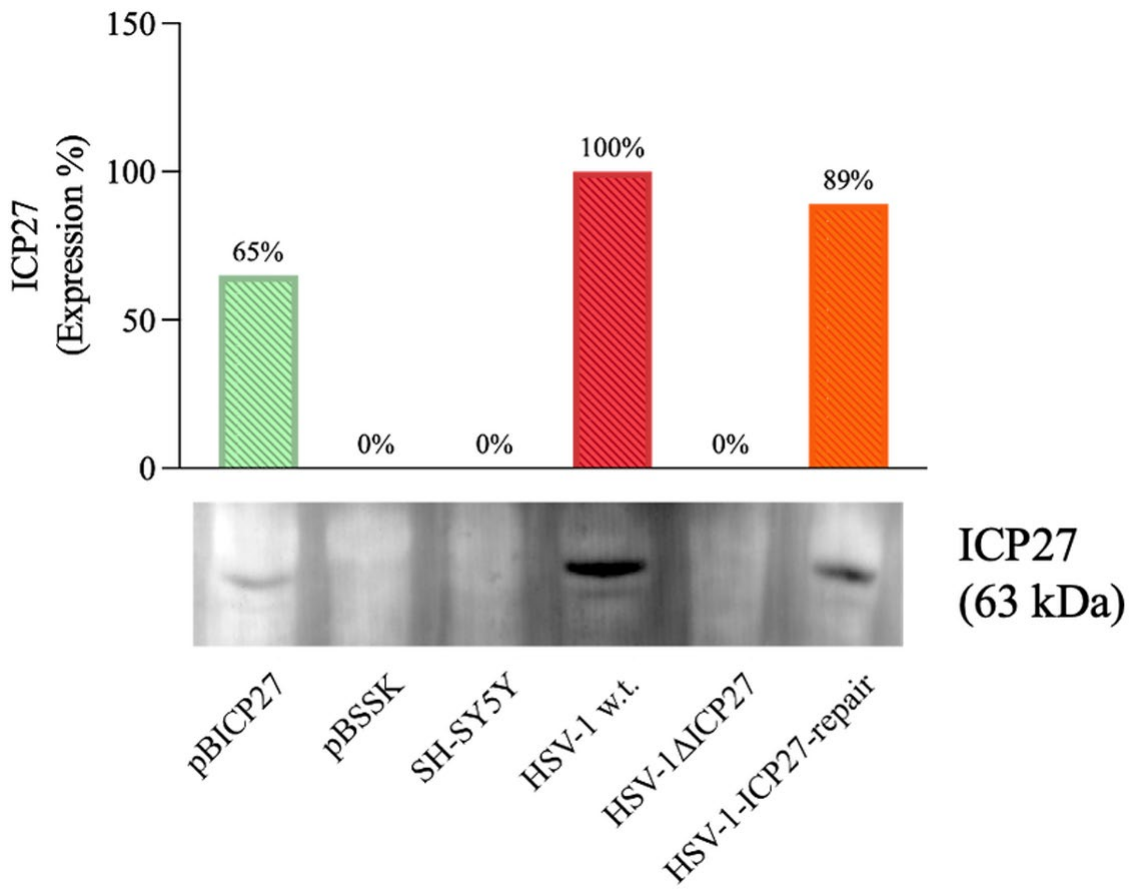
Evaluation of ICP27 protein expression. Western blot assays for ICP27 protein in SH-SY5Y cells transfected with pBICP27 and pBSSK, and infected with HSV-1 w.t., HSV-1ΔICP27, and HSV-1-ICP27-repair at 24 h post-transfection or infection. Densitometric quantification of ICP27 protein was performed using stain-free blotting, with results expressed as values relative to normalized total protein (Supplementary Figures S1, S2). Percentages indicate ICP27 levels compared to HSV-1 w.t.-infected controls (set as 100%). Total proteins (stain-free blot) and ICP27 bands were detected using the Chemidoc^™^ MP Imaging System (Bio-Rad, Segrate, Italy), and normalization was performed with Image Lab Software (version 6.0.0, Bio-Rad, Segrate, Italy).

In SH-SY5Y cells, transfection with pBICP27 plasmid, designed to express the *UL54* gene, or infection with HSV-1 w.t., or HSV-1-ICP27-repair showed strong ICP27 expression (Figure 2). Densitometric analysis revealed comparable ICP27 levels in HSV-1 w.t.- and HSV-1-ICP27-repair-infected cells, whereas no ICP27 signal was detected in HSV-1ΔICP27-infected cells or in cells transfected with the empty pBSSK vector.

Based on these results, subsequent analyses were performed using untreated, HSV-1 w.t., and HSV-1ΔICP27 conditions.

The absence of ICP27 expression in HSV-1ΔICP27-infected cells was independently confirmed by quantitative proteomics. Log₂-transformed LFQ intensities for ICP27 were 18.17 ± 0.035 in HSV-1 w.t.-infected hTert RPE-1 cells and 18.12 ± 0.035 in HSV-1 w.t.-infected SH-SY5Y cells, whereas no ICP27-derived peptides were detected in HSV-1ΔICP27 samples (Supplementary Tables 1, 2).

### 3.2 ICP27 modulates cellular metabolic activity

To evaluate the effect of ICP27 expression on cell viability, the MTT assay was performed on the two cell lines described above. Specifically, these were infected at 3 MOI with HSV-1 w.t., HSV-1ΔICP27, and HSV-1ΔICP4 for 2, 4, 8, and 24 h. The ICP4-deleted virus was included as a critical control to ensure that the observed differences were specifically attributable to ICP27 expression rather than to other immediate-early proteins, independent of viral replication competence (HSV-1 w.t. versus HSV-1ΔICP27 and HSV-1ΔICP4).

**Figure 3:**
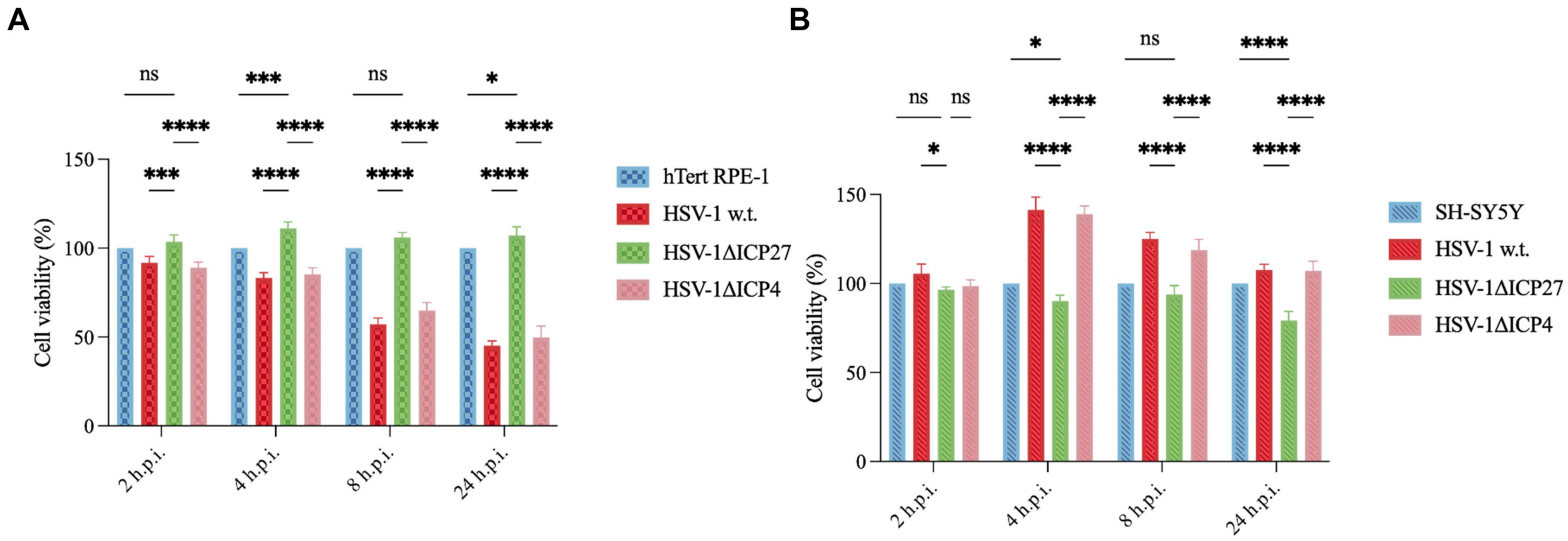
Effect of ICP27 on cell viability. **(A)** hTert RPE-1 cells and **(B)** SH-SY5Y cells were infected at a MOI of 3 with HSV-1 wild-type (w.t.), HSV-1ΔICP27, or HSV-1ΔICP4. Cell viability was measured at 2, 4, 8, and 24 h post-infection (h.p.i.) using the MTT assay. Untreated cells were included as controls. In hTert RPE-1 cells **(A)**, a time-dependent decrease in cell viability is observed in samples infected with the wild-type virus and the ICP4-deleted mutant, while cells infected with HSV-1ΔICP27 or uninfected controls show higher or comparable viability across all time points. In contrast, in SH-SY5Y cells **(B)**, HSV-1 w.t. and HSV-1ΔICP4 infection causes an increase in cell viability relative to controls and HSV-1ΔICP27-infected samples, particularly evident from 4 h.p.i. onward. Viability values are presented as mean ± SD from three independent experiments. Statistical significance was determined using two-way ANOVA (*p < 0.05, ***p < 0.001, ****p < 0.0001).

By analyzing Figure 3A, which depicts epithelial cells, it is possible to observe a time-dependent reduction in cell viability in the presence of ICP27 (HSV-1 w.t. and HSV-1ΔICP4) compared to the control (untreated cells) and to cells infected with the ICP27-deleted mutant. The latter maintain cell viability values that are either very similar to or slightly higher than those of the controls at the different time points analyzed.

A contrary trend is observed in SH-SY5Y cells (Figure 3B). Following 2 h.p.i., in which no divergence is appreciable between the different samples analyzed, from 4 h.p.i. onwards, an increase in percentage viability is observed in cells infected with the wild-type virus and the viral vector deleted for ICP4 (which expresses ICP27), and a decrease in viability for the samples infected with HSV-1ΔICP27. These observations indicate that ICP27 expression is associated with differential modulation of cellular metabolic activity depending on the cellular context. This trend is maintained at later time points, although the magnitude of the effect is reduced.

To complement the functional observations obtained from the MTT assay, proteomic analysis was performed at 8 h.p.i. allowing characterization of the main molecular changes induced by viral infection in the two cell lines studied.

**Figure 4:**
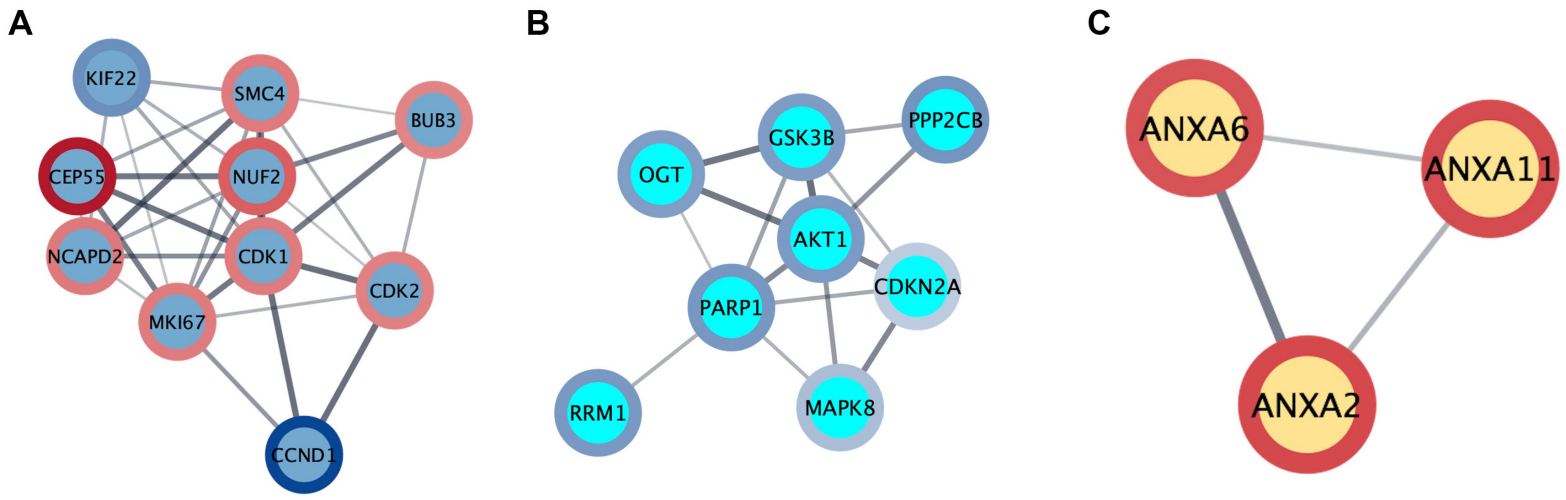
Proteomic analysis on cell viability clusters. **(A–C)** Protein–protein interaction networks were generated from proteomic data exported to the STRING database and visualized in Cytoscape. Functional clusters were identified using Markov Clustering (MCL, inflation = 3). Each node represents a protein; edge thickness corresponds to STRING interaction confidence. Node fill colors correspond to the clusters identified by the algorithm, which group proteins with common patterns of variation or functional relationships. Node border color encodes the fold change: blue indicates downregulation, red indicates upregulation. Network visualization and cluster assignment were performed in Cytoscape (version 3.10.4) using the clusterMaker2 app. Raw data are available via ProteomeXchange with identifier PXD071831. **(A)** Cluster generated from a total dataset of 317 DEPs (differentially expressed proteins) in hTert RPE-1 cells infected with HSV-1ΔICP27 vs. wild-type (8 h.p.i.): the upregulated proteins are primarily involved in cell cycle, mitotic control, and cytoskeletal organization, suggesting enrichment of cell cycle-related pathways in the absence of ICP27 and elevated cell survival as seen in the MTT assay. **(B)** Cluster of hTert RPE-1 cells infected with wild-type HSV-1 vs. control (8 h.p.i.) isolated from a total of 1,291 DEPs: downregulation of survival and stress response proteins. **(C)** Cluster generated from a total dataset of 379 DEPs in SH-SY5Y cells following HSV-1 w.t. infection, compared with untreated cells (8 h.p.i.): upregulation of annexin family proteins supports increased viability and delayed apoptosis.

In hTert RPE-1 cells infected with HSV-1ΔICP27 compared to wild-type (Figure 4A), there is a marked upregulation of proteins involved in the management of cytoarchitecture, division, and cell cycle, such as SMC4, NUF2, NCAPD2, CDK1, CDK2, CCND1, KIF22, MKI67, BUB3, and CEP55. These proteins are mainly involved in mitotic control and cell proliferation, indicating that the absence of ICP27 promotes pro-proliferative pathways and improved cell viability. This finding is consistent with the increased metabolic activity observed in the MTT assay, which shows reduced cytotoxicity and maintained survival compared to conditions of infection with wild-type HSV-1. Further confirming this trend, a direct comparison between epithelial cells infected with wild-type HSV-1 and untreated cells (Figure 4B) shows that the virus can induce an almost global downregulation of key proteins involved in survival, growth, and stress response pathways, such as RRM1, AKT1, PARP1, PPP2CB, CDKN2A, OGT, MAPK8, and GSK3B. These proteins regulate DNA replication and repair, cell cycle control, anti-apoptotic signal transduction, and mechanisms of genotoxic stress response. Their reduction reflects a global suppression of cellular defenses to promote viral replication, making cells more vulnerable to damage and cytotoxicity.

In infected SH-SY5Y cells (Figure 4C), the proteomic approach revealed markedly different regulation. Infection with wild-type HSV-1 leads to significant upregulation of annexin family members, particularly ANXA2, ANXA6, and ANXA11, which are known to be involved in the neuronal response to viral stress. ANXA2 facilitates pro-viral processes and protects against cell death, ANXA6 may act as a restrictive factor against replication and remodel the membrane, while ANXA11 is involved in cytoskeletal organization and vesicular trafficking. The induction of annexins is compatible with a strategy of cell survival and structural remodeling, preventing early apoptosis in infected neuron-like cells, as demonstrated by the increased viability observed in the MTT test compared to untreated cells. Importantly, these proteomic changes are consistent with the functional observations and suggest a link between ICP27-dependent remodeling and the observed apoptotic phenotype.

These findings indicate that ICP27 significantly influences cellular metabolic activity, exerting opposite effects depending on the cell type: reducing viability in epithelial cells while promoting survival in neuron-like cells, suggesting a context-dependent functional adaptation.

Since the results obtained using HSV-1 w.t. and HSV-1ΔICP4 are comparable, in this study the subsequent experiments reported include only untreated cells or cells infected with HSV-1 w.t. and HSV-1ΔICP27.

### 3.3 ICP27 does not interfere with cell membrane integrity

After verifying cell viability, the potential cytotoxicity of ICP27 was assessed using the LDH assay, which distinguishes between necrosis and apoptosis. The release of lactate dehydrogenase (LDH) into the extracellular medium is mainly an indicator of necrosis and acute cell injury, since apoptosis is an orderly process in which membrane integrity is preserved until apoptotic bodies form and are phagocytosed, without LDH release (Bonfoco et al., 1995; Chan et al., 2013). Therefore, both cell lines were infected with wild-type HSV-1 and HSV-1ΔICP27 for 2, 4, 8, and 24 h and LDH levels were assessed.

**Figure 5:**
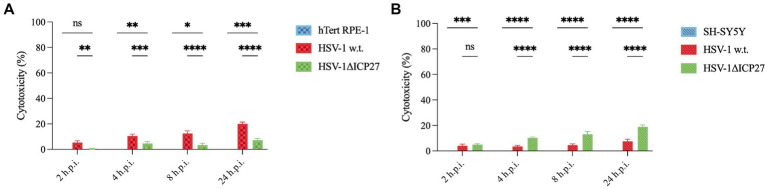
Assessment of cell membrane integrity by LDH release assay. **(A)** hTert RPE-1 cells and **(B)** SH-SY5Y cells were infected with HSV-1 w.t. or HSV-1ΔICP27 (MOI of 3) for 2, 4, 8, and 24 h. Cytotoxicity was quantified by measuring lactate dehydrogenase (LDH) release into the extracellular medium, with untreated cells as negative controls. In hTert RPE-1 cells **(A)**, infection with wild-type virus resulted in a time-dependent increase in LDH release (peaking at ~20% cytotoxicity), while cells infected with HSV-1ΔICP27 or controls maintained values below 10%. In SH-SY5Y cells **(B)**, minimal cytotoxicity was observed in wild-type HSV-1-infected samples (≤ 8%), whereas cells infected with HSV-1ΔICP27 displayed a progressive rise in cytotoxicity, reaching ~20% at 24 h post-infection. Bars represent mean ± SD from three independent experiments. Statistical significance was determined using two-way ANOVA (*p < 0.05, **p < 0.01, ***p < 0.001, ****p < 0.0001).

Figure 5A shows a time-dependent increase in cytotoxicity in epithelial cells infected with wild-type virus, reaching approximately 20% compared to the controls (untreated cells), which exhibited negligible cytotoxicity, and in cells infected with the ICP27-deleted mutant, which maintained values below 10%.

While in Figure 5B, neuron-like cells display a similar overall profile but with an inverted distribution of cytotoxicity among groups. In this case, the control cells show intact membranes without detectable cytotoxicity; cells infected with wild-type virus exhibit very low cytotoxicity levels (up to 8%), while HSV-1ΔICP27-infected cells demonstrate a time-dependent increase in cytotoxicity, peaking at about 20% at 24 h.p.i.

Although most differences were statistically significant, the low absolute cytotoxicity levels suggest that necrotic cell death does not represent the predominant mechanism under these experimental conditions. More pronounced membrane damage is evident mainly at 24 h.p.i., probably due to the involvement of other viral genes, as ICP27 activity is primarily observed during the early stages of infection when it is expressed (4–8 h after infection). Taken together, these data suggest that the observed effects on cell viability are not primarily associated with necrotic processes.

Given the low incidence of cytotoxicity observed and the nature of the damage, we therefore focused on analyzing the apoptotic pathway to investigate the mechanisms of cell death induced by infection.

### 3.4 ICP27 modulates apoptosis through the expression of executioner caspases

**Figure 6:**
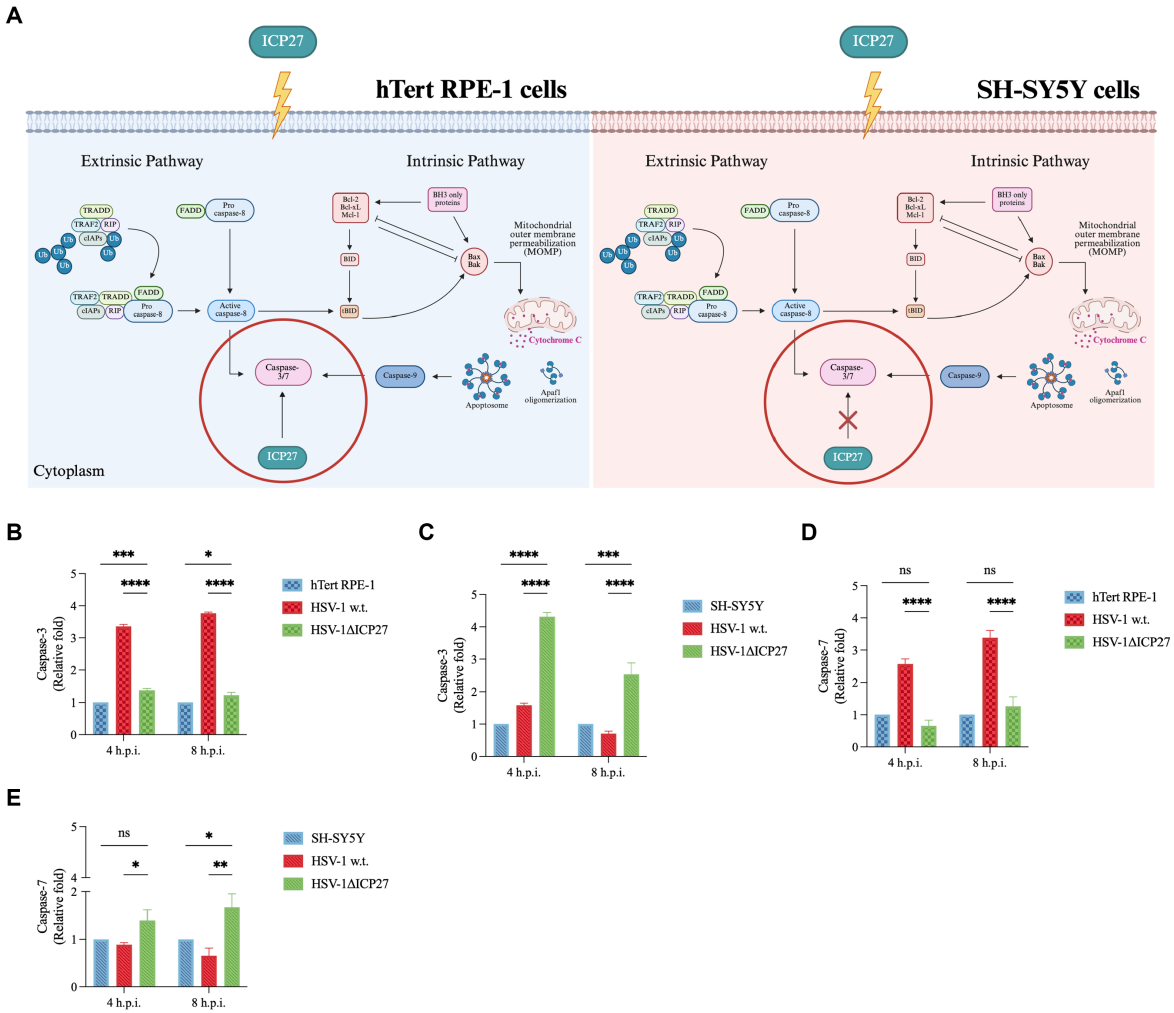
ICP27-dependent modulation of effector caspases gene expression. **(A)** Pathway focus on effector caspases: schematic representation of the intrinsic and extrinsic apoptosis pathways analyzed in both hTert RPE-1 and SH-SY5Y cell lines. The workflow illustrates key molecular events leading to programmed cell death, with the portion highlighted in red corresponding to the effector caspases (caspase-3 and caspase-7) whose regulation by ICP27 is specifically addressed in this section. **(B,D)** Gene expression levels of caspase-3 **(B)** and caspase-7 **(D)** were measured by RT-qPCR in hTert RPE-1 cells infected with HSV-1 w.t. and HSV-1ΔICP27 (MOI of 3) for 4 and 8 h.p.i.; results show increased expression in wild-type-infected samples compared to untreated cells and ICP27-deficient virus. **(C,E)** RT-qPCR quantification of caspase-3 **(C)** and caspase-7 **(E)** mRNA levels in SH-SY5Y cells infected with wild-type HSV-1 or HSV-1ΔICP27 (MOI of 3) for 4 and 8 h.p.i., revealing higher expression upon ICP27 deletion and lower levels with wild-type virus compared to untreated controls. **(B–E)** Data represent mean ± SD from three independent experiments; fold change in gene expression was calculated using the comparative 2^−ΔΔCt^ method, normalized to GAPDH and 18S rRNA, and expressed relative to untreated controls. Statistical analysis was performed using two-way ANOVA (*p < 0.05, **p < 0.01, ***p < 0.001, ****p < 0.0001).

After confirming the role of the immediate-early HSV-1 ICP27 protein in modulating cell viability in a cell type-dependent manner, and ruling out its involvement in necrosis processes, the participation of ICP27 in the regulation of apoptosis was investigated by analyzing the gene expression and activity of effector caspases, in particular caspase-3 and caspase-7 (Figure 6A). Given the active synthesis of ICP27 starting approximately 4 h post-infection and the significant variability observed at 4 and 8 h post-infection in previous experiments, the experimental investigations focused on these two time points. Epithelial and neuroblastoma cells were infected under the same conditions with wild-type virus and ICP27-deleted viral vector. Caspase gene expression was determined by real-time quantitative PCR, and caspase activity was measured using a specific bioluminescent assay.

In experiments conducted on hTert RPE-1 cells, at both time points of 4 and 8 h post-infection, the relative expression level (fold change) of effector caspases is significantly elevated in cells infected with the wild-type virus compared to untreated cells and those infected with the HSV-1ΔICP27 virus, which show expression levels similar to controls (Figures 6B,D).

SH-SY5Y cells show a distinct regulatory pattern. In this cell type, the increase in caspase-3 gene expression is more pronounced in samples infected with the ICP27-deficient virus than in controls at both 4 and 8 h post-infection (Figure 6C). Cells infected with the ICP27-expressing virus (HSV-1 w.t.), on the other hand, show slightly higher expression than controls at 4 h.p.i., with a consequent reduction 8 h after infection. Similarly, caspase-7 shows a trend of similar magnitude (Figure 6E), with expression levels generally lower than those observed for caspase-3.

**Figure 7:**
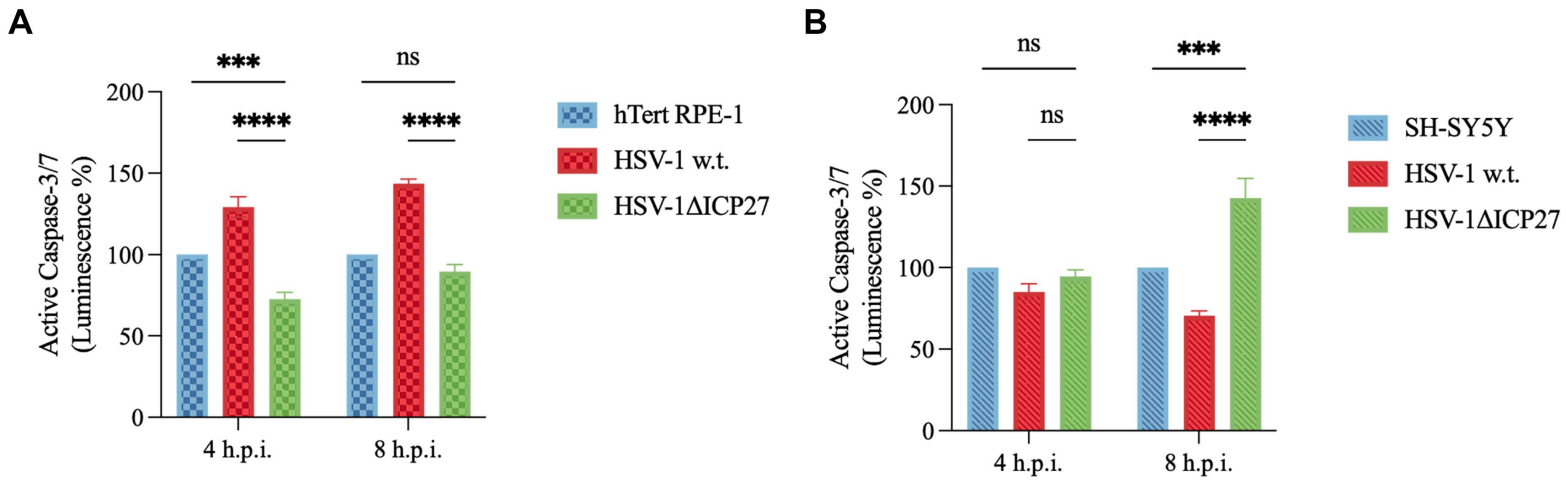
ICP27-dependent modulation of effector caspases activity. **(A,B)** Caspase-3/7 enzymatic activity was quantified by bioluminescence assay in epithelial **(A)** and neuron-like **(B)** cells infected under the same conditions, confirming elevated activity after wild-type infection in hTert RPE-1 cells, and a peak at 8 h.p.i. in SH-SY5Y cells only for ICP27-deleted virus. Data represent mean ± SD from three independent experiments; statistical analysis was performed using two-way ANOVA (***p < 0.001, ****p < 0.0001).

Furthermore, to confirm the gene expression data, a bioluminescent assay was performed to quantify the enzymatic activity of the caspase-3/7 complex. As shown in Figure 7A, a significantly higher percentage of luminescence, indicative of high enzymatic activity, is observed in hTert RPE-1 cells infected with the wild-type virus compared to the controls. Viral vector lacking ICP27-infected cells show lower caspase activity than the control at 4 h post-infection, then return to baseline levels at 8 h.p.i.

As for SH-SY5Y cells, Figure 7B shows no significant changes in caspase-3/7 activity at 4 h, compared to a peak in activity at 8 h.p.i. in samples infected with HSV-1ΔICP27, relative to both controls and wild-type-infected cells, in which a decrease in effector caspases activity is observed.

These results suggest that, in epithelial cells, the presence of ICP27 increases the expression and activity of caspases-3 and -7, enzymes responsible for the irreversible execution of programmed cell death. Conversely, in neuron-like cells, ICP27 appears to exert an inhibitory role, reducing caspase expression and activity, and thus is associated with reduced activation of apoptotic signaling pathways.

### 3.5 ICP27 plays a significant role in the intrinsic pathway of apoptosis

**Figure 8:**
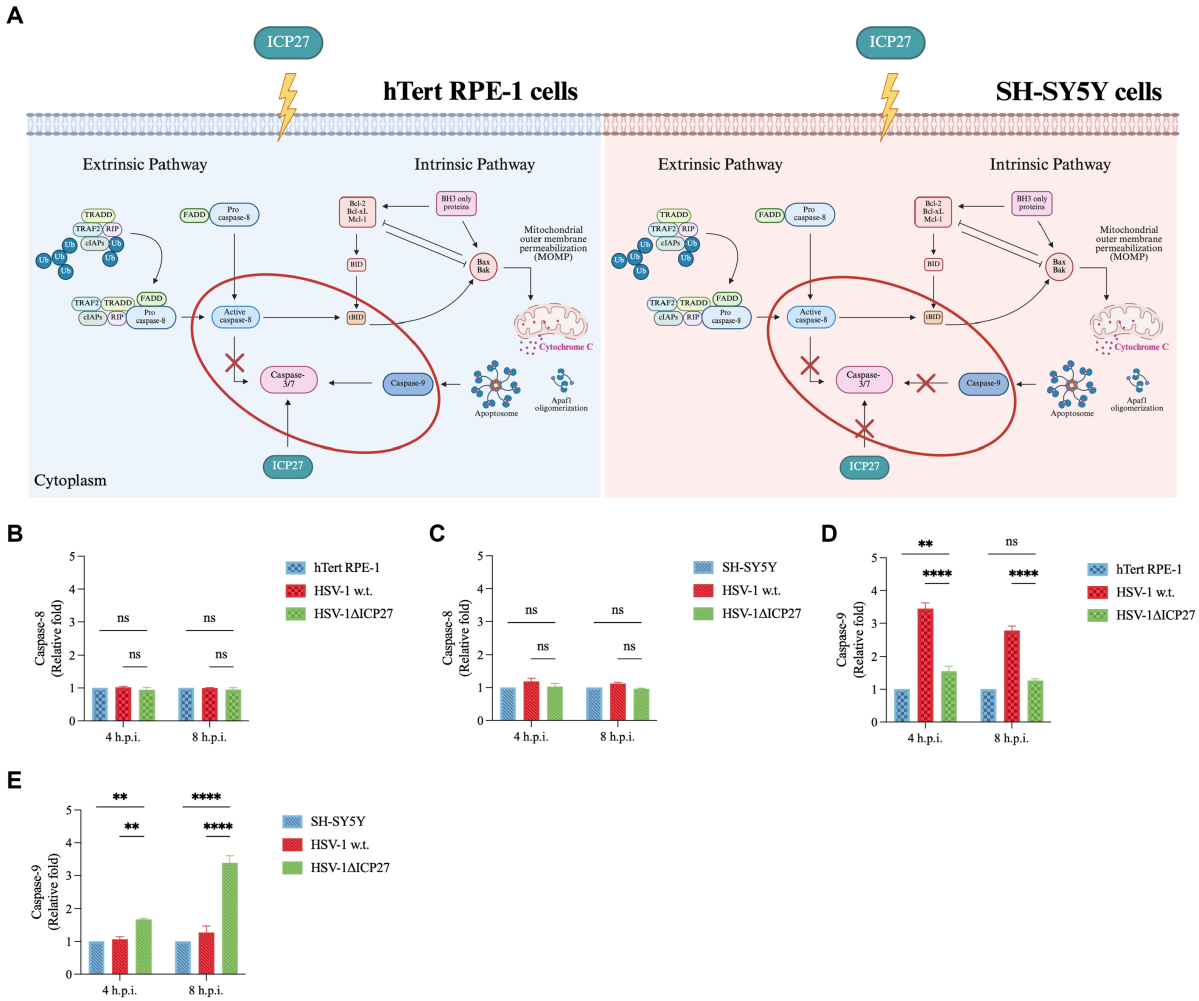
ICP27 selectively modulates the intrinsic apoptotic pathway. **(A)** Pathway focus on initiator caspases: graphical summary of the experimental workflow illustrating intrinsic and extrinsic apoptosis pathways in hTert RPE-1 and SH-SY5Y cell lines. The portion of the pathway corresponding to initiator caspases is emphasized, with a focus on caspase-8 (extrinsic) and caspase-9 (intrinsic). **(B,C)** RT-qPCR analyses of caspase-8 gene expression in hTert RPE-1 **(B)** and SH-SY5Y **(C)** cells infected with HSV-1 w.t. or HSV-1ΔICP27 (MOI of 3) at 4 and 8 h.p.i. reveal no significant change compared to controls. **(D,E)** Caspase-9 mRNA levels measured by RT-qPCR in hTert RPE-1 **(D)** and SH-SY5Y **(E)** cells infected under identical conditions (MOI of 3, 4 and 8 h.p.i.), showing marked upregulation in wild-type HSV-1-infected epithelial cells and in HSV-1ΔICP27-infected neuron-like cells, respectively, with distinct time-dependent patterns. **(B–E)** Data are presented as mean ± SD from three independent experiments; fold change in gene expression was calculated using the comparative 2^−ΔΔCt^ method, normalized to GAPDH and 18S rRNA, and expressed relative to untreated controls. Statistical analyses were performed using two-way ANOVA (**p < 0.01, ****p < 0.0001).

Considering that programmed cell death can be induced by internal or external stimuli, the possible involvement of ICP27 in the modulation of initiator caspases, caspase-8 and caspase-9, which activate the extrinsic and intrinsic pathways of apoptosis, respectively, was analyzed (Figure 8A). To this end, hTert RPE-1 and SH-SY5Y cell lines were infected with wild-type HSV-1 and the ICP27-deficient mutant virus (HSV-1ΔICP27) at a multiplicity of infection (MOI) of 3. Gene expression was evaluated by real-time PCR at both 4 and 8 h post-infection, while protein analysis by western blot was performed only at 8 h post-infection.

The results show that caspase-8 gene expression remains unchanged across all experimental conditions in both cell lines (Figures 8B,C), indicating no effect on the extrinsic pathway.

In contrast, caspase-9 expression is significantly increased in epithelial cells (Figure 8D) infected with wild-type HSV-1 compared to the controls, while in HSV-1ΔICP27-infected cells, a transient increase is observed at 4 h, which returns to baseline by 8 h post-infection.

In SH-SY5Y neuron-like cells, caspase-9 gene expression increases progressively, reaching a peak at 8 h post-infection following HSV-1ΔICP27 infection, exceeding levels observed in both controls and wild-type virus samples (Figure 8E).

**Figure 9:**
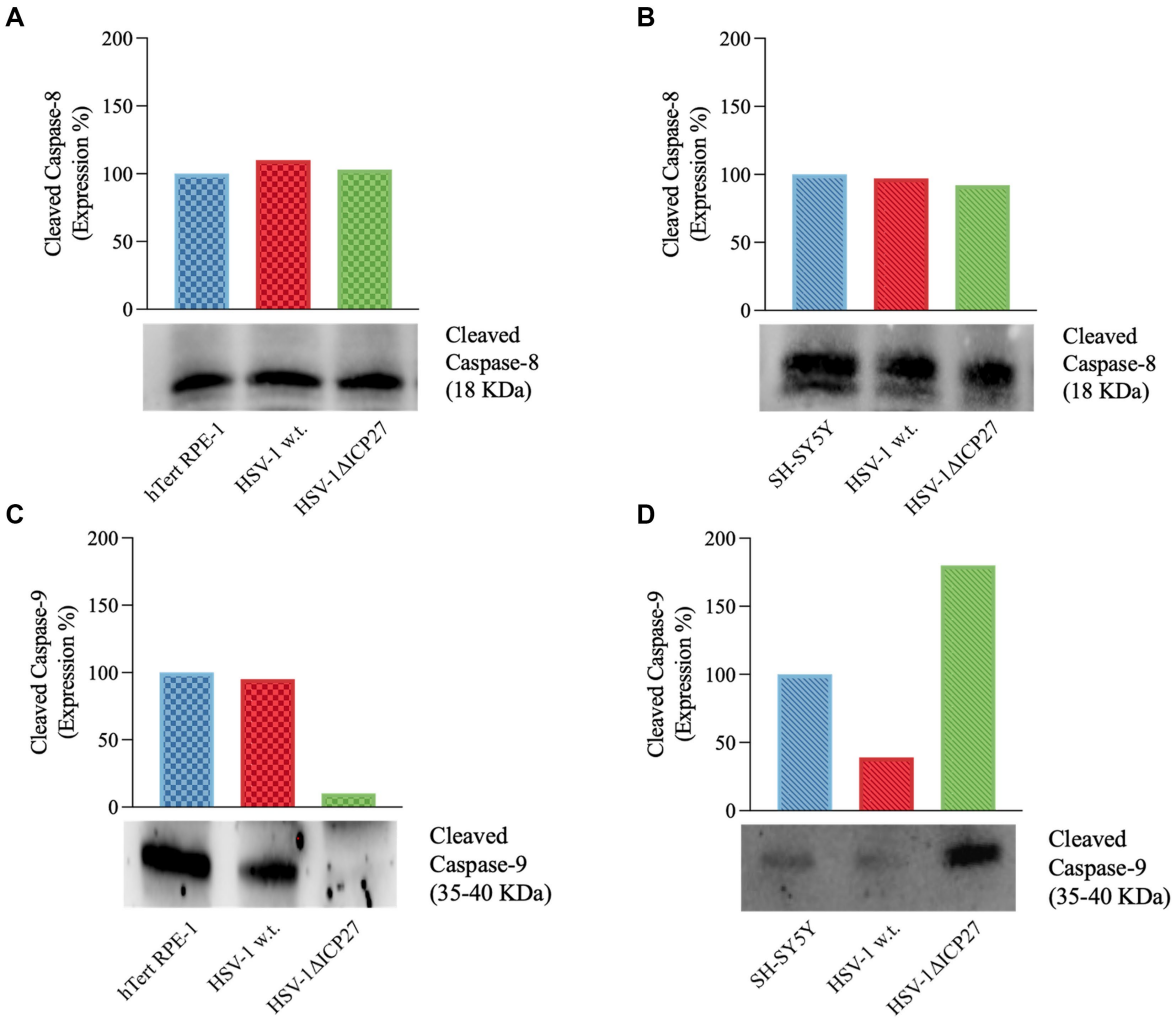
Protein expression of caspase-8 and caspase-9. **(A,B)** Western blot analyses of cleaved caspase-8 protein levels at 8 h.p.i. in hTert RPE-1 **(A)** and SH-SY5Y **(B)** cells infected with HSV-1 w.t. or HSV-1ΔICP27 (MOI of 3) confirm the absence of ICP27-dependent modulation in the extrinsic pathway. **(C,D)** Cleaved caspase-9 protein levels at 8 h.p.i. in epithelial **(C)** and neuron-like **(D)** cells, infected as described above, demonstrate unchanged expression in wild-type HSV-1-infected hTert RPE-1 cells, a decrease in HSV-1ΔICP27-infected hTert RPE-1 sample, and an increase in SH-SY5Y cells upon HSV-1ΔICP27 infection. **(A–D)** Densitometric quantifications of cleaved caspase-8 and -9 levels were performed using stain-free blotting, with results expressed as values relative to normalized total protein (Supplementary Figures S3–S10). Percentages indicate cleaved caspase-8 and -9 levels compared to untreated cells (set as 100%). Total proteins (stain-free blot) and band signals were detected using the Chemidoc^™^ MP Imaging System (Bio-Rad, Segrate, Italy), and normalization was performed with Image Lab software (Bio-Rad, Segrate, Italy). Western blot data are representative of independent experiments showing consistent trends.

Protein analysis at 8 h.p.i. confirms these data by showing constant levels of cleaved caspase-8 under different conditions (Figures 9A,B).

Figure 9C highlights the proteolytic activation of Caspase-9 in hTert RPE-1 cells. While constant levels of the cleaved (active) form are maintained between control and HSV-1 w.t.-infected cells, a sharp decrease in caspase-9 cleavage is observed in the absence of ICP27, suggesting a direct or indirect role for this viral protein in triggering the mitochondrial cascade in epithelial cells.

On the contrary, neuron-like cells exhibit a selective increase in cleaved caspase-9 expression following HSV-1ΔICP27 infection and a decrease with wild-type virus infection, relative to control (Figure 9D).

These results therefore suggest a selective involvement of caspase-9-associated signaling, thereby contributing to the regulation of the intrinsic apoptotic pathway, while exerting no significant effect on the caspase-8-mediated extrinsic pathway.

### 3.6 ICP27 affects ROS production and mitochondrial functionality

**Figure 10:**
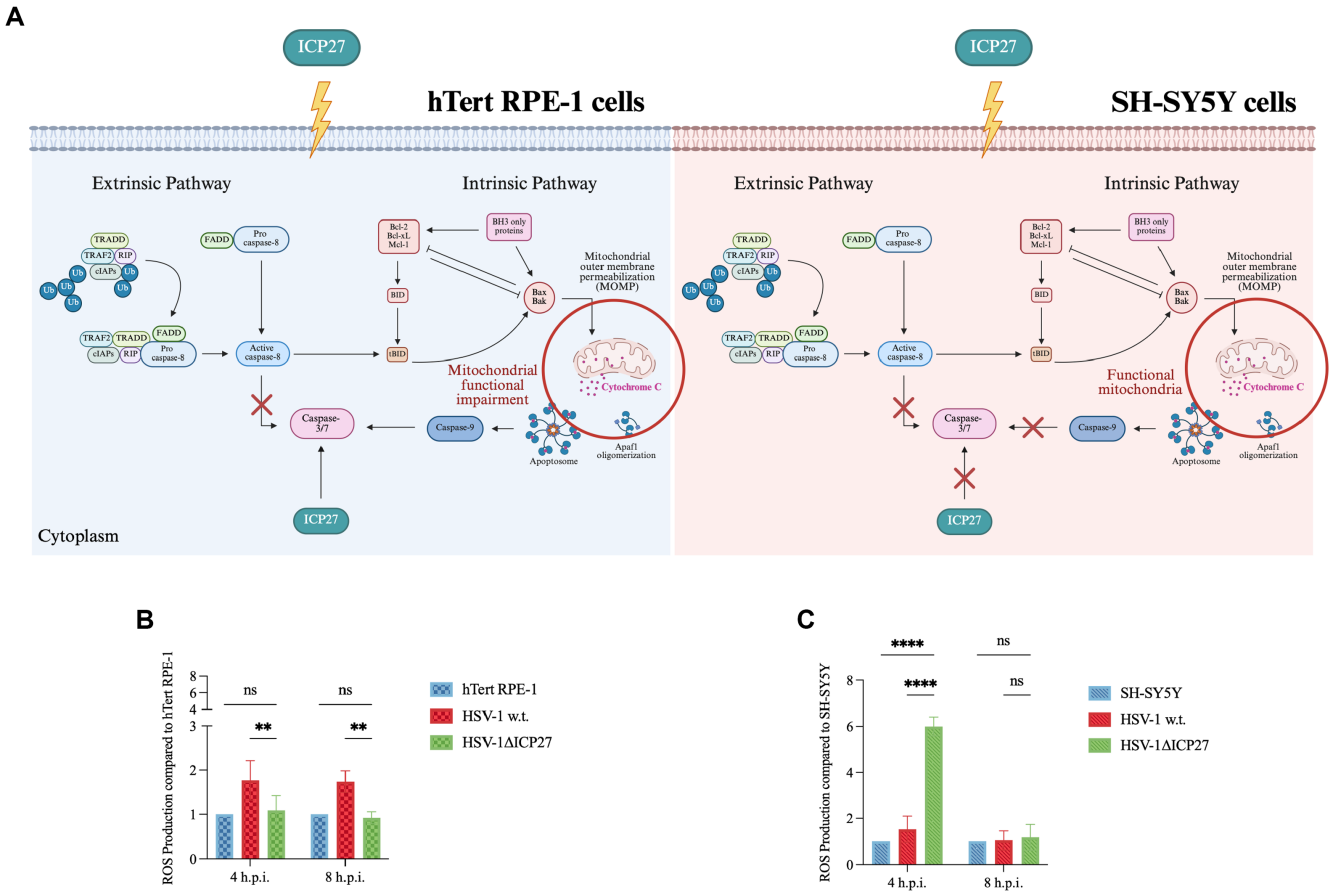
ICP27 regulates ROS production in a cell-context-dependent manner (epithelial vs. neuron-like cells). **(A)** Pathway focus on mitochondrial homeostasis and oxidative stress. Conceptual diagram illustrating the role of ICP27 on mitochondrial metabolism and intrinsic apoptosis in hTert RPE-1 (left) and SH-SY5Y (right) cells: in epithelial cells, ICP27 promotes oxidative stress and transient mitochondrial hyperpolarization; in neuron-like cells, the opposite effect occurs. **(B,C)** Flow cytometry of intracellular reactive oxygen species (ROS) production in hTert RPE-1 and SH-SY5Y cells infected with wild-type HSV-1 or HSV-1ΔICP27 (MOI of 3, 4, and 8 h.p.i.). In epithelial cells **(B)**, wild-type infection induces a significant increase in ROS compared to control and HSV-1ΔICP27 infection. In SH-SY5Y cells **(C)**, the ROS peak is observed only at 4 h.p.i. with HSV-1ΔICP27; it does not persist at 8 h.p.i. All values are presented as means ± SD of at least three independent experiments; statistical analysis for flow cytometry data was performed using two-way ANOVA (**p < 0.01, ****p < 0.0001).

Since the intrinsic apoptotic pathway is also defined as the mitochondrial pathway, it was evaluated whether the viral protein ICP27 could play a role in the intracellular production of reactive oxygen species (ROS), given that mitochondria are one of the main cellular compartments responsible for their generation, and because these molecules are crucial signals that compromise cellular integrity (Wu and Bratton, 2013; Redza-Dutordoir and Averill-Bates, 2016). At the same time, the possible influence of ICP27 on mitochondrial functionality was analysed (Figure 10A).

Both cell lines, hTert RPE-1 and SH-SY5Y, were infected with wild-type HSV-1 and HSV-1ΔICP27 at 3 MOI for 4 and 8 h, and the parameters described above were subsequently analysed by flow cytometry.

In epithelial cell samples (Figure 10B), intracellular ROS production is significantly increased under HSV-1 w.t. infection compared to untreated controls and samples infected with the ICP27-deficient mutant, which maintained basal ROS levels.

In contrast, in neuron-like cells (Figure 10C), a peak in ROS production is observed 4 h post-infection, but only in cells infected with HSV-1ΔICP27, compared to the other conditions. This difference dissipates by 8 h, as ROS level in HSV-1ΔICP27-infected cells is return to baseline, suggesting the possible activation of cellular compensation or adaptation mechanisms that restore redox balance after initial stress.

Together, these observations suggest that ICP27 is associated with differential regulation of oxidative stress depending on the cellular context.

To determine whether the observed variations in reactive oxygen species (ROS) production were associated with changes in mitochondrial function, mitochondrial membrane potential (Δψm) was measured using TMRM and normalized to mitochondrial mass. This ratio provides an estimate of mitochondrial functional status independent of mitochondrial content.

**Figure 11:**
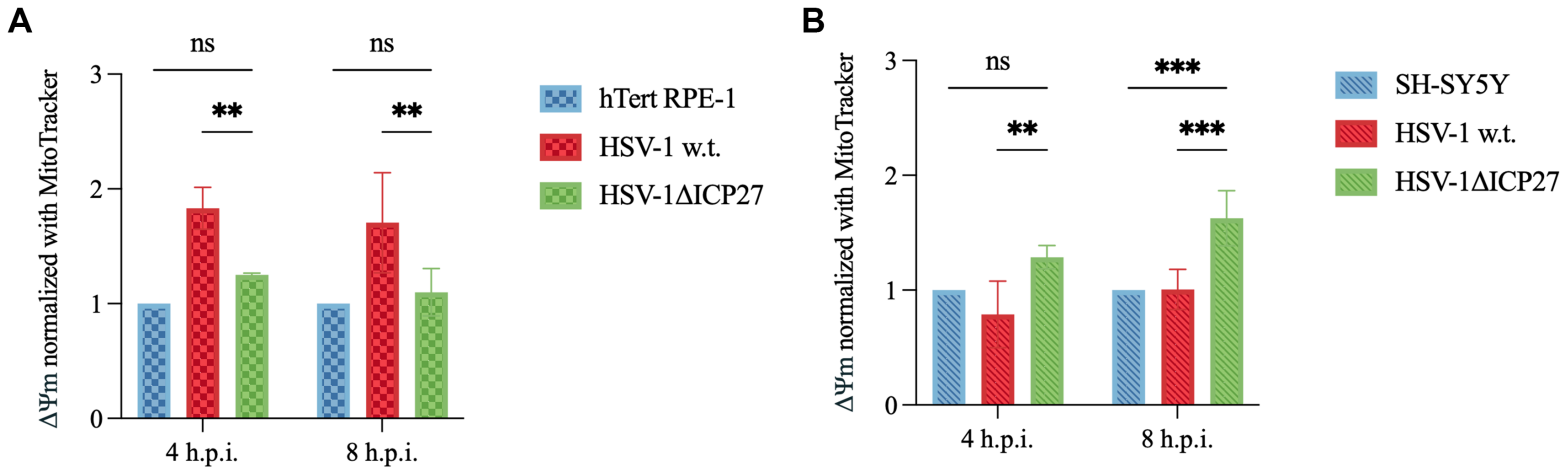
ICP27 regulates mitochondrial functionality cell-context-dependent manner in epithelial (hTert RPE-1) versus neuron-like (SH-SY5Y) cells. **(A,B)** Membrane potential/mitochondrial quantity ratio (TMRM/MitoTracker) was assessed by flow cytometry in the two cell lines infected under the same experimental conditions. In hTert RPE-1 cells **(A)**, wild-type HSV-1 induces an increase in the ratio at 4 and 8 h.p.i., suggesting changes in mitochondrial membrane potential; in SH-SY5Y cells **(B)**, the same occurs only for HSV-1ΔICP27 at 8 h.p.i. All values are presented as means ± SD of at least three independent experiments; statistical analysis for flow cytometry data was performed using two-way ANOVA (**p < 0.01, ***p < 0.001).

In hTert RPE-1 cell line (Figure 11A), an increase in this ratio is observed at 4 and 8 h post-infection in HSV-1 w.t.-infected cells, suggesting alterations in mitochondrial function.

In SH-SY5Y cells (Figure 11B), no substantial differences are found between the experimental conditions at 4 h.p.i.; however, at 8 h.p.i., a significant increase in the membrane potential/mitochondrial mass ratio is observed only in samples infected with HSV-1ΔICP27.

**Figure 12:**
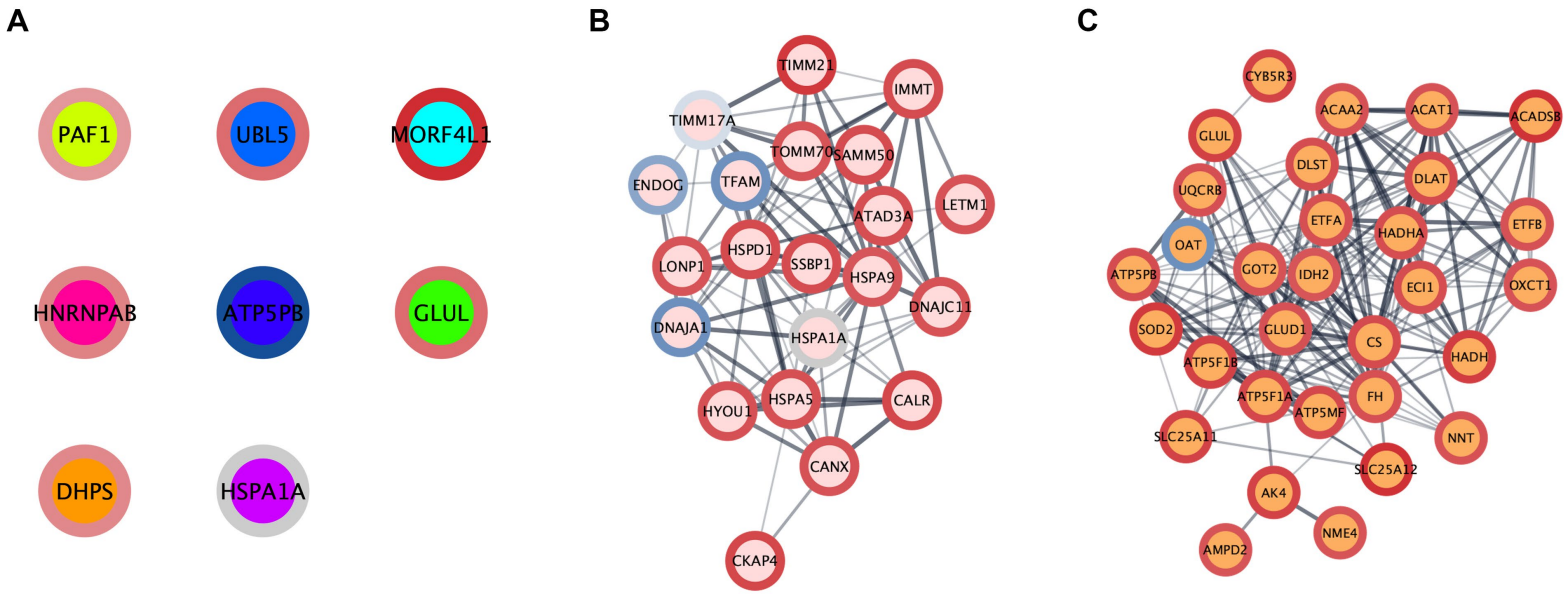
Proteomic analysis on SH-SY5Y mitochondrial clusters. **(A–C)** Protein–protein interaction networks were generated from proteomic data exported to the STRING database and visualized in Cytoscape. Functional clusters were identified using Markov Clustering (MCL, inflation = 3). Each node represents a protein; edge thickness corresponds to STRING interaction confidence. Node fill colors correspond to the clusters identified by the algorithm, which group proteins with common patterns of variation or functional relationships. Node border color encodes the fold change: blue indicates downregulation, red indicates upregulation. Network visualization and cluster assignment were performed in Cytoscape (version 3.10.4) using the clusterMaker2 app. Raw data are available via ProteomeXchange with identifier PXD071831. **(A)** Complete protein network consisting of the total dataset of 8 DEPs identified in SH-SY5Y cells infected with the ICP27-deleted mutant vs. wild-type (8 h.p.i.): downregulation of ATP5PB reduces the ability of mitochondria to dissipate membrane potential, promoting a state of hyperpolarization. **(B)** Protein network isolated from a total dataset of 379 DEPs in neuron-like cells infected with HSV-1 w.t. (8 h.p.i.) compared to control, showing a strong positive regulation (red border) of proteins associated with the cell cycle, mitotic control, and cytoskeleton. **(C)** Mitochondrial proteins network identified within the same dataset of 379 DEPs (HSV-1 w.t. vs. control in SH-SY5Y cells at 8 h.p.i.): includes complexes involved in mitochondrial homeostasis, proteostasis, import, and assembly of membrane proteins, together with proteins involved in oxidative phosphorylation and energy metabolism.

This increase in mitochondrial membrane potential can be explained by the downregulation of ATP5PB, as shown in the proteomic comparison of SH-SY5Y cells infected with the ICP27-deleted virus and HSV-1 w.t. (Figure 12A). ATP5PB is a subunit of mitochondrial ATP synthase responsible for ATP synthesis by exploiting the proton gradient generated by the respiratory chain. Its downregulation reduces the ability to dissipate membrane potential via ATP synthesis, thereby promoting “hyperpolarization” of the mitochondrial membrane (Forkink et al., 2014; García-Aguilar and Cuezva, 2018). In contrast, additional protein clusters of interest were identified in wild-type-infected SH-SY5Y cells (Figures 12B,C). The first cluster (pink) includes numerous proteins involved in mitochondrial homeostasis, protein folding, and cellular stress response. Notably, it includes key components such as TIMM21, TIMM17A, TOMM70, ATAD3A, LETM1, SAMM50, and IMMT, which are involved in mitochondrial protein import, membrane assembly, and maintenance of mitochondrial structure. In addition, SSBP1, LONP1, and TFAM are associated with the maintenance, replication, and transcription of mitochondrial DNA, as well as the proteolysis of abnormal proteins within the mitochondrion. The second cluster (orange) consists almost exclusively of mitochondrial proteins involved in energy metabolism, the Krebs cycle, oxidative phosphorylation (including ATP5F1A, ATP5F1B, ATP5PB, ATP5MF), amino acid and fatty acid metabolism. The red border highlights their upregulation, suggesting an adaptive response aimed at optimizing protein and mitochondrial stress management, and enhancing mitochondrial oxidative capacity and ATP synthesis. These proteins are associated with mitochondrial homeostasis and may contribute to the preservation of mitochondrial integrity. This finding is corroborated by preserved mitochondrial function: ROS levels and TMRM/mitochondrial mass ratios are comparable to those observed in untreated cells, indicating that energy production, morphology, and mitochondrial proteostasis remain essentially unchanged following infection with wild-type virus.

These results indicate that ICP27 induces a sustained elevation of ROS production in epithelial cells, resulting in compromised mitochondrial function. In contrast, in neuron-like cells, the same functional impairment appears to occur only in the absence of ICP27, suggesting a context-dependent effect.

### 3.7 ICP27 regulates the Bax/Bcl-2 ratio and the apoptotic propensity index

**Figure 13:**
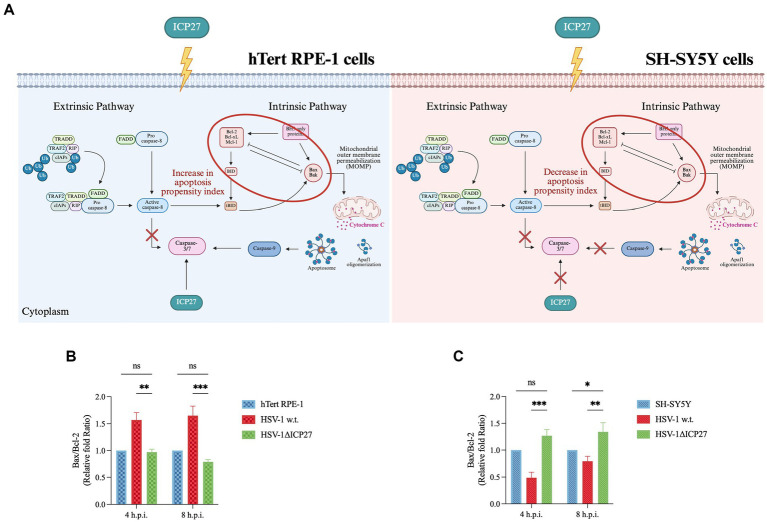
ICP27-dependent modulation of Bax/Bcl-2 ratio and apoptotic propensity. **(A)** Pathway focus on apoptosis propensity index (Bax/Bcl-2 ratio): schematic representation of the intrinsic and extrinsic apoptotic pathways in hTert RPE-1 and SH-SY5Y cells, highlighting the effect of ICP27 on the Bax/Bcl-2 ratio. The area marked in red represents the mitochondrial pathway steps specifically investigated in this experimental section. **(B,C)** Quantification of Bax/Bcl-2 mRNA ratio by RT-qPCR in hTert RPE-1 and SH-SY5Y cells infected with HSV-1 w.t. or HSV-1ΔICP27 for 4 and 8 h at MOI of 3. In hTert RPE-1 cells **(B)**, the Bax/Bcl-2 ratio significantly exceeds 1 only in wild-type-infected samples at both time points, while in SH-SY5Y cells **(C)** the ratio is above unity exclusively in HSV-1ΔICP27-infected samples. Bars indicate mean ± SD of three independent experiments; statistical analysis performed by two-way ANOVA (*p < 0.05, **p < 0.01, ***p < 0.001).

To conclude the investigation into how ICP27 regulates intrinsic apoptosis, the last critical node of mitochondrial cell death control was designed to address the Bax/Bcl-2 ratio and its impact on cellular propensity for apoptosis (Figure 13A). This experiment constitutes the concluding step of the pathway analysis, enabling the integration of gene expression patterns with functional measures of apoptosis.

Apoptosis is strictly regulated by numerous genes that control key points in programmed cell death. Among these, the Bcl-2 family of proteins plays a central role, modulating the transduction of apoptotic signals at the mitochondrial level. In particular, Bcl-2 exerts an anti-apoptotic action by blocking various death stimuli, including the release of cytochrome c, an event necessary for the activation of effector caspases. On the contrary, Bax is the main pro-apoptotic member of the family: its expression promotes mitochondrial permeabilization and the progression of the intrinsic pathway (Donovan and Cotter, 2004; Brunelle and Letai, 2009; Edlich, 2018). The functional balance between the dimeric forms of Bax and Bcl-2 influences cellular susceptibility to apoptotic stimuli. Therefore, the Bax/Bcl-2 ratio is a critical index of apoptotic propensity: a value greater than 1 is associated with increased sensitivity to cell death, while a value less than 1 correlate with greater resistance (Raisova et al., 2001).

Having previously demonstrated that ICP27 selectively modulates the intrinsic pathway of apoptosis, we further investigated this by evaluating the ratio between Bax and Bcl-2 gene expression in hTert RPE-1 and SH-SY5Y cell lines after infection with wild-type HSV-1 and ICP27-deleted mutant (MOI of 3) at 4 and 8 h.p.i.

As shown in Figure 13B representing hTert RPE-1 cells, the Bax/Bcl-2 ratio is significantly higher than 1 exclusively in cells infected with wild-type HSV-1 at both 4 and 8 h post-infection, compared to untreated cells and those infected with HSV-1ΔICP27, for which values close to the control are observed.

In contrast, in SH-SY5Y cells (Figure 13C), the Bax/Bcl-2 ratio exceeds 1 following infection with HSV-1ΔICP27, while it remains below unity in cells exposed to the wild-type virus compared to the respective controls.

Taken together, these data suggest that ICP27 is linked to the modulation of the Bax/Bcl-2 ratio, potentially influencing the cellular propensity toward programmed cell death in a cell type-dependent manner.

## 4 Discussion

Herpes simplex virus type 1 represents a paradigmatic model of virus-host interaction, in which the balance between lytic replication, persistence, and reactivation depends on the capacity of viral proteins to modulate host signaling pathways (Nicoll et al., 2012; Whitley and Baines, 2018; Steiner and Kennedy, 1993). Understanding how HSV-1 reshapes cellular stress and death responses is essential for clarifying the molecular determinants that govern viral dissemination and long-term persistence (Guo et al., 2022; Caproni et al., 2024).

Immediate-early proteins play a central role in initiating the viral transcriptional cascade and reprogramming host cell functions (Sandri-Goldin, 2011; Tunnicliffe et al., 2015; Malik et al., 2012; Aubert and Blaho, 1999; Tang et al., 2016; Soto-Machuca et al., 2025). Among them, ICP27, encoded by the *UL54* gene, is a multifunctional regulator involved in viral mRNA processing, nuclear export, and host gene expression remodeling (Sandri-Goldin, 2008). While previous studies have suggested both pro- and anti-apoptotic roles for ICP27 (Aubert and Blaho, 2001; Gillis et al., 2009; Kim et al., 2017), its contribution to intrinsic apoptotic signaling across different cellular contexts remains incompletely defined.

In the present study, we demonstrate that ICP27 modulates intrinsic apoptotic signaling in a cell type-dependent manner. In hTert RPE-1 epithelial cells, ICP27 expression is associated with reduced metabolic activity, increased activation of caspase-3/7, selective upregulation of caspase-9, elevated Bax/Bcl-2 ratio, sustained ROS production, and altered mitochondrial membrane potential. Collectively, these findings indicate that ICP27 is associated with enhanced activation of mitochondrial apoptotic signaling in epithelial cells, in agreement with previous reports linking ICP27 to Bax translocation and mitochondrial signaling (Gillis et al., 2009; Kim et al., 2017). Notably, caspase-8 expression and cleavage remained unchanged, supporting a preferential involvement of the intrinsic rather than the extrinsic pathway, although additional mechanistic validation would be required to fully dissect upstream signaling events (You et al., 2017).

In contrast, SH-SY5Y neuron-like cells exhibited an opposite regulatory pattern. While recognizing that their neuroblastoma origin and undifferentiated state do not fully recapitulate primary neuronal physiology, these cells remain a useful and widely employed neuron-like model. In this setting, ICP27 expression correlated with preserved metabolic activity, reduced caspase-3/7 activation, limited caspase-9 induction, and maintenance of mitochondrial redox balance. The absence of ICP27 led to increased intrinsic apoptotic activation, elevated ROS production at early time points, and alterations in mitochondrial membrane potential. These observations are consistent with previous evidence suggesting a cytoprotective role of ICP27 during early stages of infection (Sanfilippo et al., 2004; Aubert et al., 2001) and support the notion that HSV-1 modulates apoptotic response in a cell-dependent manner (You et al., 2017).

Proteomic analyses further support this differential regulation. In epithelial cells, deletion of ICP27 was associated with enrichment of cell cycle-related proteins, whereas wild-type infection induced downregulation of host survival pathways, consistent with extensive viral remodeling of cellular functions during lytic infection (Aubert and Blaho, 1999; Tang et al., 2016). In neuron-like cells, wild-type infection induced upregulation of mitochondrial and stress response proteins, including components involved in protein import, mitochondrial DNA maintenance, and oxidative phosphorylation. This proteomic profile is compatible with mitochondrial homeostasis preservation under conditions of ICP27 expression and aligns with previous evidence demonstrating HSV-1-induced remodeling of mitochondrial DNA, morphology, and bioenergetic function during infection (Leclerc et al., 2024). Importantly, these proteomic changes are consistent with the functional data, supporting either a pro-apoptotic or pro-survival cellular state depending on ICP27 expression and cellular context.

These findings are compatible with a model in which ICP27 limits intrinsic apoptotic activation in neuron-like cells, possibly through previously described interactions with 14–3-3θ and Bax (You et al., 2017; Kim et al., 2017). In this context, ICP27 expression correlates with reduced progression toward cytolysis and enhanced cell survival. However, this interpretation should be considered within the limitations of the model, as SH-SY5Y cells do not fully reproduce the complexity of neuronal latency or *in vivo* neuronal responses (Kim et al., 2017; Aubert and Blaho, 1999).

The divergent effects observed in epithelial and neuron-like cells may reflect the distinct requirements of HSV-1 infection in peripheral tissues versus neuronal reservoirs (Wilson and Mohr, 2012; Cliffe and Knipe, 2008). In epithelial cells, where productive lytic replication predominates, enhanced intrinsic apoptosis may facilitate viral dissemination (Aubert and Blaho, 2001). In contrast, within neuronal environments, excessive apoptotic activation would be detrimental to long-term persistence. The ability of ICP27 to differentially modulate apoptotic signaling may therefore contribute to the broader plasticity of HSV-1 infection across distinct cellular microenvironments (Guo et al., 2022; Tang et al., 2019).

Future studies employing primary human sensory neurons, induced pluripotent stem cell-derived neuronal systems, or in vivo infection models will be necessary to confirm whether the context-dependent role of ICP27 observed here is maintained under physiologically relevant conditions. Furthermore, while our results provide a consistent picture of apoptotic signaling, further biochemical characterization including Cytochrome c release, PARP cleavage, and functional inhibition studies will be essential to provide a definitive mechanistic demonstration of the pathway’s activation.

Nevertheless, the concordance among multiple independent readouts, including caspase activation, Bax/Bcl-2 ratio, ROS production, mitochondrial functional parameters, and proteomic remodeling, strongly supports a cell-context-dependent role of ICP27 in intrinsic apoptosis.

In conclusion, our findings indicate that ICP27 differentially regulates intrinsic apoptotic signaling according to the cellular environment, promoting mitochondrial apoptotic activation in epithelial cells while restricting it in neuron-like cells. This functional plasticity provides further insight into how HSV-1 adapts to distinct microenvironments and underscores the relevance of ICP27 as a regulator of virus-host interaction dynamics (Guo et al., 2022; Sandri-Goldin, 2008; Rutan and Dembowski, 2026).

## Data Availability

The mass spectrometry proteomics data have been deposited to the ProteomeXchange Consortium via the PRIDE (Perez-Riverol 2025) partner repository with the dataset identifier PXD071831. All relevant data is contained within the article: the original contributions presented in the study are included in the article/Supplementary material, further inquiries can be directed to the corresponding author.
