# Supplementary material for: Life-death trade-offs: HSV-1 ICP27 differentially modulates intrinsic apoptotic signaling in epithelial and neuron-like cells
Source: Front Microbiol. 2026 Jun 15;17:1849379. doi: 10.3389/fmicb.2026.1849379 (PMC13311084; doi:10.3389/fmicb.2026.1849379)
Supplement: Supplementary file 1 [file Data_Sheet_1.docx]

Supplementary Material

# Supplementary Figures and Tables

## Supplementary Figures


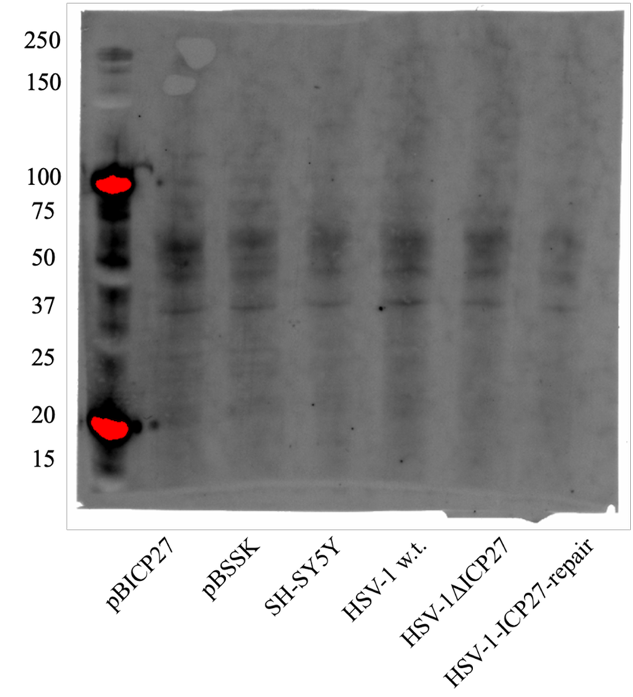


**Supplementary Figure 1.** Stain-free total protein blot of SH-SY5Y cells transfected with pBICP27 or pBSSK and infected with HSV-1 wild-type, HSV-1ΔICP27, or HSV-1 repair at 24 h post-transfection/infection. Total protein detection was performed using the ChemiDoc™ MP Imaging System (Bio-Rad, Segrate, Italy). Band normalization (Figure 2) was conducted with Image Lab software (version 6.0.0, Bio-Rad, Segrate, Italy). Molecular weight marker: Precision Plus Protein™ All Blue Prestained Protein Standards (Bio-Rad, Segrate, Italy).


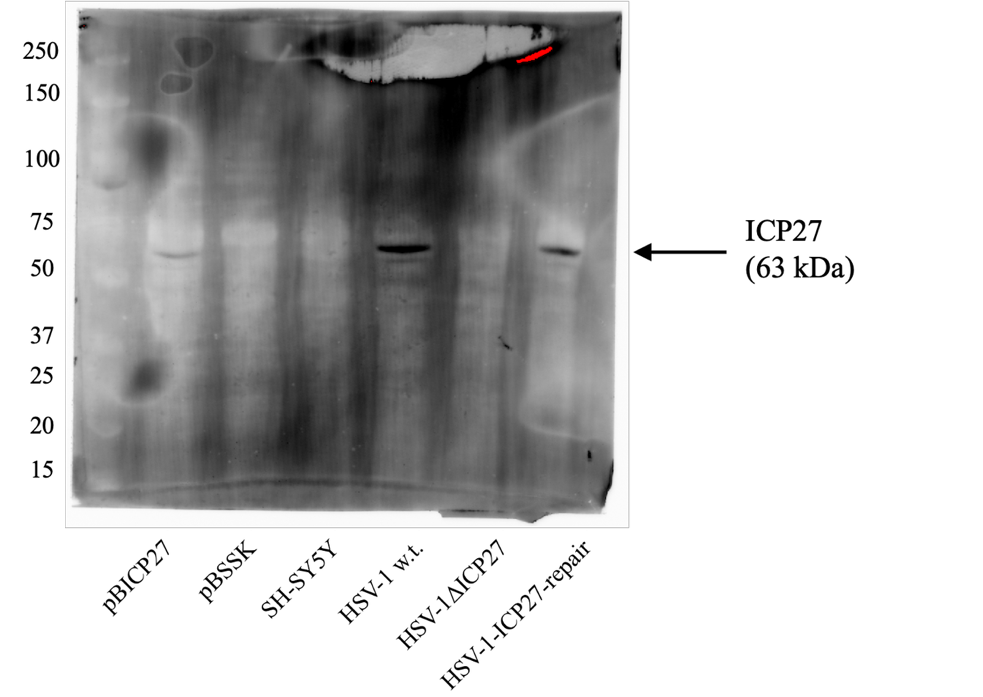


**Supplementary Figure 2.** Figure 2 uncropped. Western blot analysis of ICP27 (63 kDa) in SH-SY5Y cells transfected with pBICP27 or pBSSK, and infected with HSV-1 wild-type, HSV-1ΔICP27, or HSV-1 repair at 24 h post-transfection/infection. Molecular weight marker: Precision Plus Protein™ All Blue Prestained Protein Standards (Bio-Rad, Segrate, Italy).


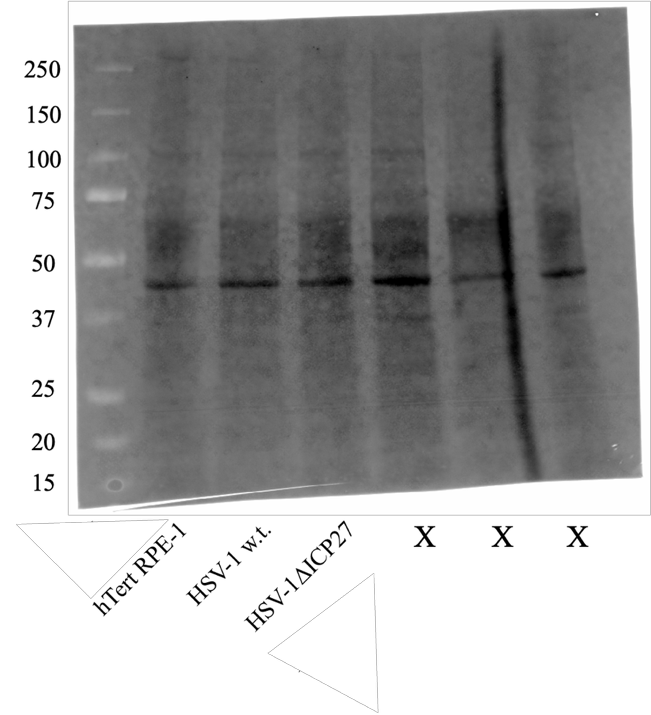


**Supplementary Figure 3.** Stain-free total protein blot of untreated hTert RPE-1, HSV-1 w.t., and HSV-1ΔICP27 infected cells (MOI of 3) at 8 h.p.i. Total protein detection was performed using the ChemiDoc™ MP Imaging System (Bio-Rad, Segrate, Italy). Band normalization (Figure 9A) was conducted with Image Lab software (version 6.0.0, Bio-Rad, Segrate, Italy). Molecular weight marker: Precision Plus Protein™ All Blue Prestained Protein Standards (Bio-Rad, Segrate, Italy).


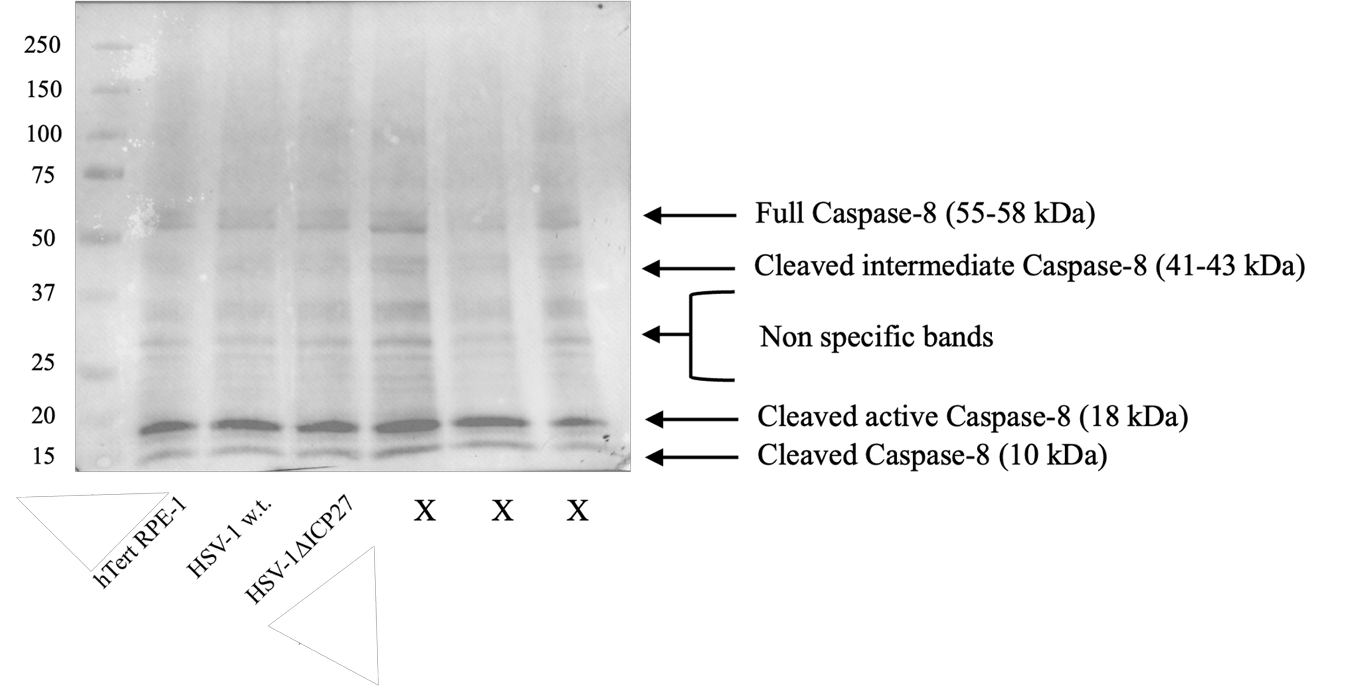


**Supplementary Figure 4.** Figure 9A uncropped. Western blot analysis of cleaved caspase-8 (18 kDa) in untreated hTert RPE-1, HSV-1 w.t., and HSV-1ΔICP27 infected cells (MOI of 3) at 8 h.p.i.

Molecular weight marker: Precision Plus Protein™ All Blue Prestained Protein Standards (Bio-Rad, Segrate, Italy).


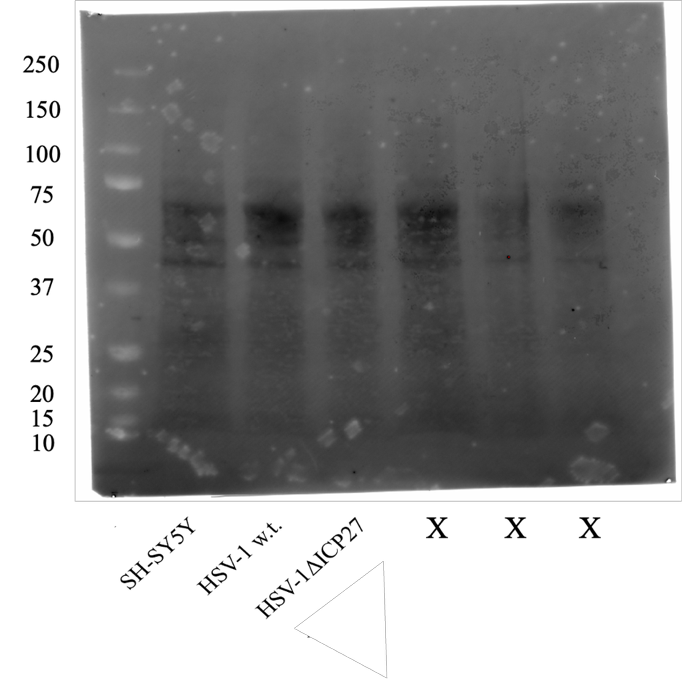


**Supplementary Figure 5.** Stain-free total protein blot of untreated SH-SY5Y, HSV-1 w.t., and HSV-1ΔICP27 infected cells (MOI of 3) at 8 h.p.i. Total protein detection was performed using the ChemiDoc™ MP Imaging System (Bio-Rad, Segrate, Italy). Band normalization (Figure 9B) was

conducted with Image Lab software (version 6.0.0, Bio-Rad, Segrate, Italy). Molecular weight marker: Precision Plus Protein™ All Blue Prestained Protein Standards (Bio-Rad, Segrate, Italy).


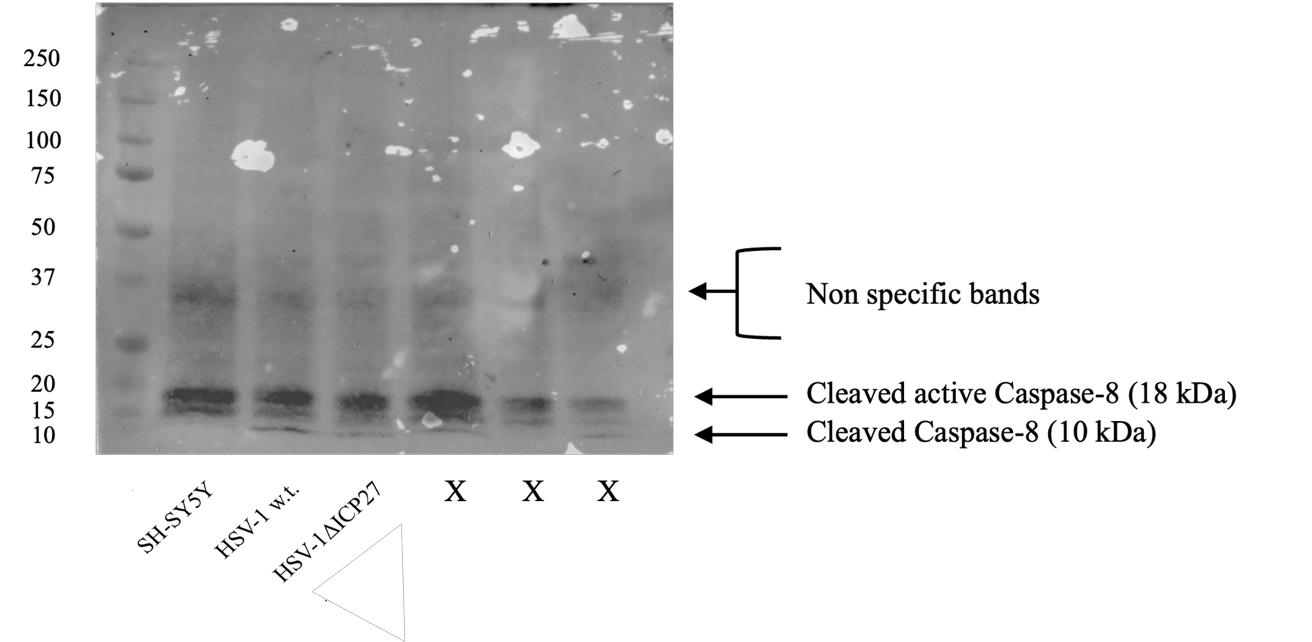
**Supplementary Figure 6.** Figure 9B uncropped. Western blot analysis of cleaved caspase-8 (18 kDa) in untreated SH-SY5Y, HSV-1 w.t., and HSV-1ΔICP27 infected cells (MOI of 3) at 8 h.p.i.

Molecular weight marker: Precision Plus Protein™ All Blue Prestained Protein Standards (Bio-Rad, Segrate, Italy).


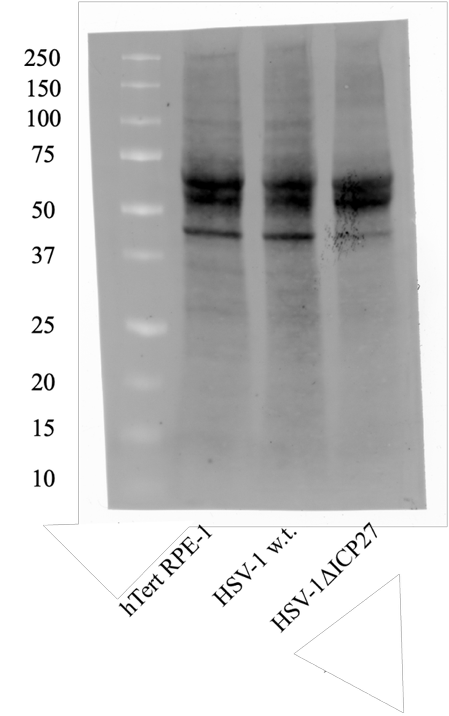


**Supplementary Figure 7.** Stain-free total protein blot of untreated hTert RPE-1, HSV-1 w.t., and HSV-1ΔICP27 infected cells (MOI of 3) at 8 h.p.i. Total protein detection was performed using the ChemiDoc™ MP Imaging System (Bio-Rad, Segrate, Italy). Band normalization (Figure 9C) was

conducted with Image Lab software (version 6.0.0, Bio-Rad, Segrate, Italy). Molecular weight marker: Precision Plus Protein™ All Blue Prestained Protein Standards (Bio-Rad, Segrate, Italy).


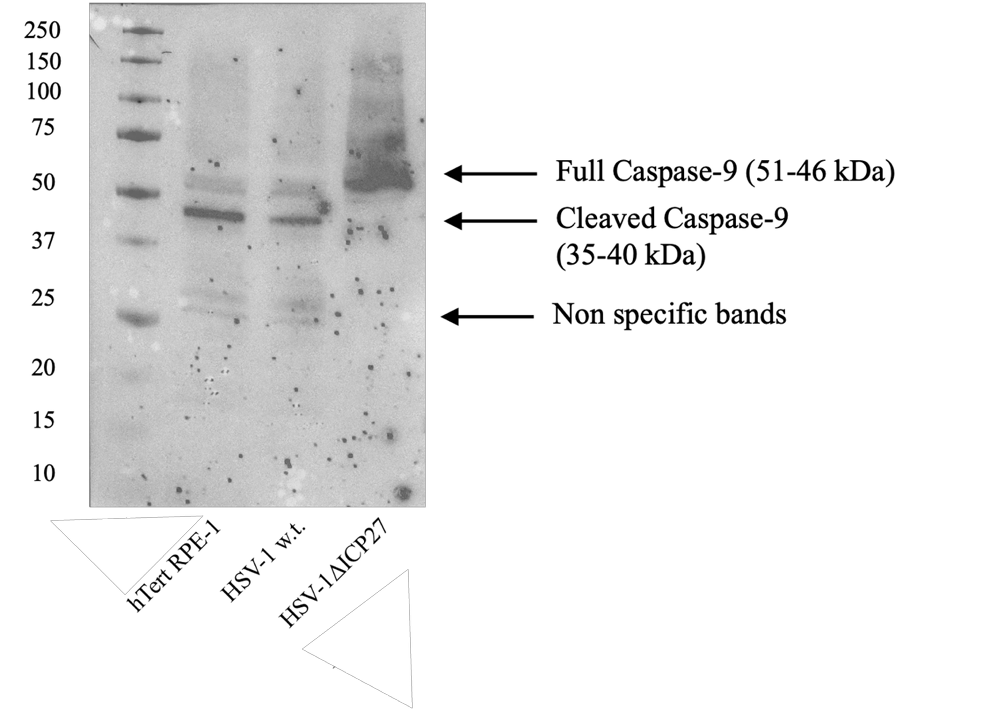
**Supplementary Figure 8.** Figure 9C uncropped. Western blot analysis of cleaved caspase-9 (35-40 kDa) in untreated hTert RPE-1, HSV-1 w.t., and HSV-1ΔICP27 infected cells (MOI of 3) at 8 h.p.i.

Molecular weight marker: Precision Plus Protein™ All Blue Prestained Protein Standards (Bio-Rad, Segrate, Italy).


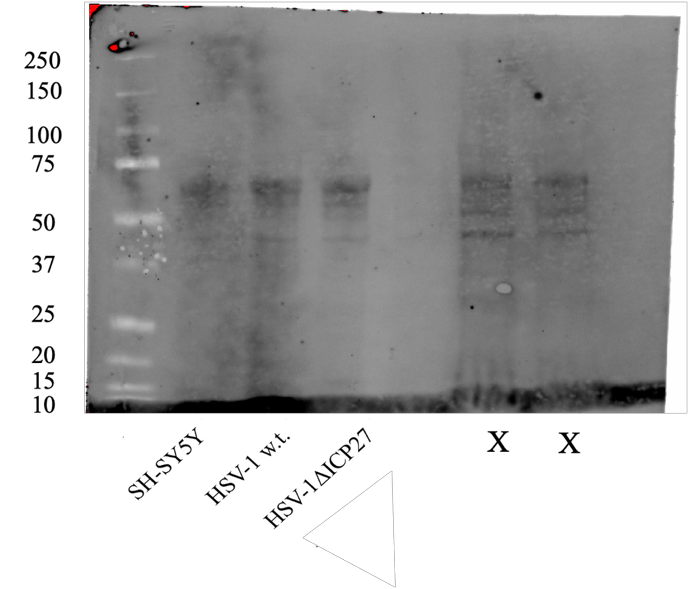


**Supplementary Figure 9.** Stain-free total protein blot of untreated SH-SY5Y, HSV-1 w.t., and HSV-1ΔICP27 infected cells (MOI of 3) at 8 h.p.i. Total protein detection was performed using the ChemiDoc™ MP Imaging System (Bio-Rad, Segrate, Italy). Band normalization (Figure 9D) was

conducted with Image Lab software (version 6.0.0, Bio-Rad, Segrate, Italy). Molecular weight marker: Precision Plus Protein™ All Blue Prestained Protein Standards (Bio-Rad, Segrate, Italy).


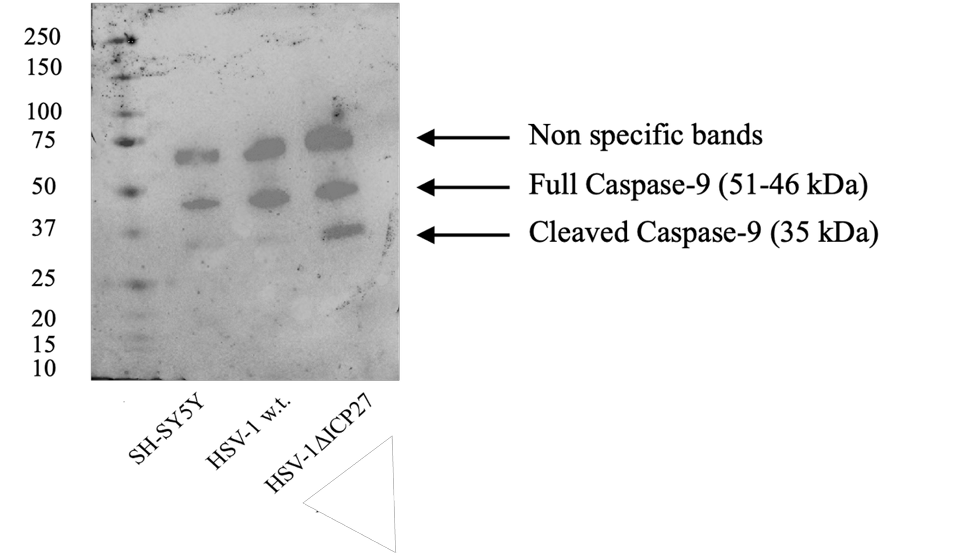
**Supplementary Figure 10.** Figure 9D uncropped. Western blot analysis of cleaved caspase-9 (35 kDa) in untreated SH-SY5Y, HSV-1 w.t., and HSV-1ΔICP27 infected cells (MOI of 3) at 8 h.p.i. Molecular weight marker: Precision Plus Protein™ All Blue Prestained Protein Standards (Bio-Rad, Segrate, Italy).

## Supplementary Tables

**Supplementary Table 1.** Quantification of the ICP27 protein across experimental conditions of HSV-1 w.t.- or HSV-1ΔICP27-infected hTert RPE-1 cells. Values are reported as log2-transformed LFQ intensities. NaN (not a number) values correspond to missing quantification, reflecting the absence of detectable ICP27 peptide signals in the corresponding samples.

| Protein | hTert RPE-1 HSV-1 w.t. | hTert RPE-1 HSV-1 w.t. | hTert RPE-1 HSV-1 w.t. | hTert RPE-1 HSV-1ΔICP27 | hTert RPE-1 HSV-1ΔICP27 | hTert RPE-1 HSV-1ΔICP27 |
| --- | --- | --- | --- | --- | --- | --- |
| ICP27 | 18,2052 | 18,1707 | 18,1661 | NaN | NaN | NaN |

**Supplementary Table 2.** Quantification of the ICP27 protein across experimental conditions of HSV-1 w.t.- or HSV-1ΔICP27-infected SH-SY5Y cells. Values are reported as log2-transformed LFQ intensities. NaN (not a number) values correspond to missing quantification, reflecting the absence of detectable ICP27 peptide signals in the corresponding samples.

| Protein | SH-SY5Y HSV-1 w.t. | SH-SY5Y HSV-1 w.t. | SH-SY5Y HSV-1 w.t. | SH-SY5Y HSV-1ΔICP27 | SH-SY5Y HSV-1ΔICP27 | SH-SY5Y HSV-1ΔICP27 |
| --- | --- | --- | --- | --- | --- | --- |
| ICP27 | 18,1386 | 18,1225 | 18,0875 | NaN | NaN | NaN |
